# Decoding the gene regulatory network of endosperm differentiation in maize

**DOI:** 10.1038/s41467-023-44369-7

**Published:** 2024-01-02

**Authors:** Yue Yuan, Qiang Huo, Ziru Zhang, Qun Wang, Juanxia Wang, Shuaikang Chang, Peng Cai, Karen M. Song, David W. Galbraith, Weixiao Zhang, Long Huang, Rentao Song, Zeyang Ma

**Affiliations:** 1 State Key Laboratory of Maize Bio-breeding, Frontiers Science Center for Molecular Design Breeding, Joint International Research Laboratory of Crop Molecular Breeding, National Maize Improvement Center, College of Agronomy and Biotechnology, China Agricultural University, Beijing, 100193 China; 2 Sanya Institute of China Agricultural University, Sanya, 572025 China; 3 Hainan Yazhou Bay Seed Laboratory, Sanya, 572025 China; 4 Department of Biology, Trinity College of Arts and Sciences, Duke University, Durham, NC 27708 USA; 5 School of Plant Sciences and Bio5 Institute, University of Arizona, Tucson, AZ 85721 USA

**Keywords:** Plant development, Plant breeding, Genome-wide analysis of gene expression

## Abstract

The persistent cereal endosperm constitutes the majority of the grain volume. Dissecting the gene regulatory network underlying cereal endosperm development will facilitate yield and quality improvement of cereal crops. Here, we use single-cell transcriptomics to analyze the developing maize (*Zea mays*) endosperm during cell differentiation. After obtaining transcriptomic data from 17,022 single cells, we identify 12 cell clusters corresponding to five endosperm cell types and revealing complex transcriptional heterogeneity. We delineate the temporal gene-expression pattern from 6 to 7 days after pollination. We profile the genomic DNA-binding sites of 161 transcription factors differentially expressed between cell clusters and constructed a gene regulatory network by combining the single-cell transcriptomic data with the direct DNA-binding profiles, identifying 181 regulons containing genes encoding transcription factors along with their high-confidence targets, Furthermore, we map the regulons to endosperm cell clusters, identify cell-cluster-specific essential regulators, and experimentally validated three predicted key regulators. This study provides a framework for understanding cereal endosperm development and function at single-cell resolution.

## Abstract (web summary)

The cereal endosperm constitutes most of the grain by volume. Here the authors use single-cell analysis of maize developing endosperm to decode gene regulatory networks that likely control endosperm growth and offer a framework for crop improvement.

## Introduction

Maize (*Zea mays*) endosperm is an important food source for humans and animals^1–3^ and an excellent model for developmental and molecular studies, given its relatively large size and greater repertoire of cell types compared to those of other crop species, such as wheat (*Triticum aestivum*), rice (*Oryza sativa*) and barley (*Hordeum vulgare*)^3–6^. After double fertilization, early maize endosperm development includes a multinucleate coenocyte stage, a cellularization stage to form the cellular endosperm, and then the stage of differentiation into distinct cell types^6–8^. In early differentiation, maize endosperm comprises the immature starchy endosperm (SE), aleurone layer (AL), basal endosperm transfer layer (BETL) and embryo-surrounding region (ESR) cell types; the subaleurone (SA), conducting zone (CZ), basal intermediate zone (BIZ) and endosperm adjacent to scutellum (EAS) arise in late differentiation^4,7,9^. Based on morphological observations, 6–8 days after pollination (DAP) is a critical time window bridging the early and late differentiation stages^7^. Once the main cell types are established, maize endosperm undergoes intensive cell proliferation and transitions to the grain-filling stage^10^.

Despite this detailed body of knowledge, the regulatory mechanisms underlying endosperm cell fate determination and differentiation remain largely unknown. Transcriptomic studies have provided spatiotemporal molecular characterization of the major maize endosperm cell types^9,11–15^, but a high-resolution map of endosperm cells at differentiation stage has not been established, hindering our understanding of the heterogeneous cell populations and of the underlying biological processes. A cohort of transcription factors (TFs) involved in maize endosperm development has been identified^16^, but a comprehensive regulatory network governing the formation and maintenance of the different cell types, as well as their master regulators, have yet to be defined.

To build a comprehensive map of the transcriptional landscape and a gene regulatory network of endosperm differentiation in maize and to describe its transcriptional complexity, we used single-cell transcriptomics (scRNA-seq) and optimized DNA affinity purification sequencing with PCR-amplified genomic DNA library (ampDAP-seq) to generate a transcriptome atlas and large-scale TF-DNA-binding profiles, respectively. Based on the reconstituted high-confidence gene regulatory network, we also identified essential regulons for each cell type. Finally, we provide an online interface for exploring these datasets (https://www.maize-endosperm.cn/).

## Results

### Construction of a single-cell transcriptome atlas of maize endosperm

**Fig. 1:**
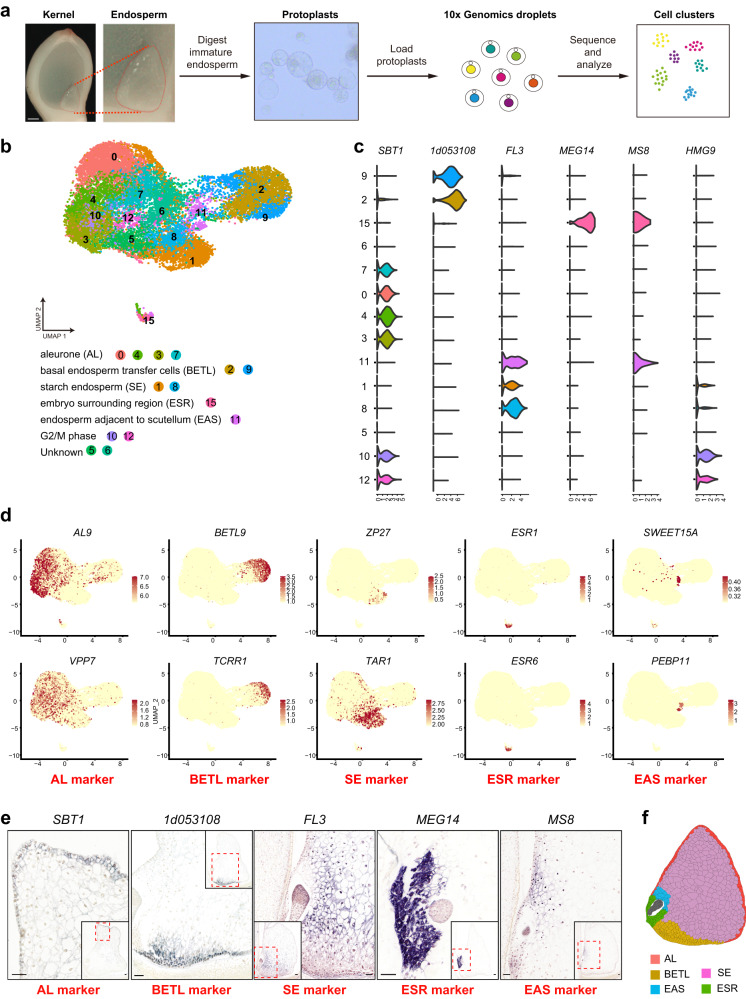
Construction of a single-cell transcriptomic atlas for developing maize endosperm. **a** Overview of the endosperm scRNA-seq and cluster annotation workflow using scRNA-seq libraries generated using protoplasts isolated from 6 and 7 DAP immature maize endosperms; scale bars, 1 mm. **b** UMAP visualization of maize endosperm cells displaying 14 putative clusters. Each dot represents a single cell, color-coded by cell clusters. **c** Violin plots showing the expression patterns of representative cell-type marker genes in 14 cell clusters. **d** UMAP plots of marker genes revealing the identities of cell clusters. *AL9*, *VPP7*, AL markers; *BETL9*, *TCRR1*, BETL markers; *ZP27*, *TAR1*, SE markers; *ESR1*, *ESR6*, ESR markers; *SWEET15A*, *PEBP11*, EAS markers. Color scale represents normalized expression levels. **e** mRNA in situ hybridization results of marker genes validated cell-cluster identities. *SBT1*, AL marker; *1d053108*, BETL marker; *FL3*, SE marker; *MEG14*, ESR marker; *MS8*, EAS marker; scale bars, 50 μm. Experiments were repeated three times yielding similar results. **f** Spatial distribution of cell clusters in a longitudinal section sketch of the maize endosperm.

To generate a single-cell atlas from differentiating endosperm, we harvested maize endosperm tissues at 6 and 7 DAP and digested them into protoplasts. We constructed four scRNA-seq libraries (one sample from 6 DAP and three replicates from 7 DAP) using the 10x Genomics Chromium platform, which were sequenced using Illumina technologies (Fig. 1a, Supplementary Fig. 1, Supplementary Data 1). We profiled 17,022 individual cells that passed quality control and detected the expression of 25,365 genes, which was close to the number (30,050) detected by bulk RNA sequencing (RNA-seq) of the same samples (Supplementary Fig. 1, Supplementary Data 1). On average, we detected the expression of 2005 genes per cell. The global gene-expression profiles were highly correlated between undigested endosperm cells and protoplasts, as well as between the scRNA-seq and bulk RNA-seq of protoplasts (*r* = 0.95 and 0.83, respectively; Supplementary Fig. 1a, b). The quality control plots indicated high reproducibility between biological replicates (Supplementary Fig. 1c, d).

To identify distinct cell populations based on the gene-expression profiles, we aggregated scRNA-seq data from all samples using the Harmony algorithm. We clustered all sequenced cells using the Seurat package in an unsupervised manner, obtaining 18 clusters ranging from 82 to 2484 cells each (Supplementary Figs. 2–5, Supplementary Data 2). The effects of cell-cycle heterogeneity on cell clustering were mitigated using a list of cell-cycle-related genes^17–19^ (Supplementary Data 3) and the standard Seurat pipeline (See methods). Most of the cells having cell-cycle heterogeneity were grouped into clusters (Supplementary Fig. 2). The numbers of expressed genes per cell from different clusters were similar (Supplementary Fig. 3b). Quality inspections such as Pearson’s correlation analysis between cells and clusters illustrate the high reliability of our clustering (Supplementary Fig. 4). Differential expression analysis to identify the genes more strongly expressed in each cluster revealed specific expression patterns of the genes representative of each cluster, which included some cell-type marker genes reported for maize endosperm: e.g., the *Embryo-surrounding region* 2 (*ESR2*) in ESR cells^20^ (Supplementary Fig. 5a, Supplementary Data 4). We annotated the identity of cell clusters using a manually curated list of reported marker genes for kernel cell types^9,12,15^ and mRNA in situ hybridization (ISH) assays of newly identified cluster-representative transcripts (Fig. 1, Supplementary Figs. 6 and7). We then removed cells from further analysis that represented contaminating tissues (cluster 17 for embryo and cluster 14 for nucellus) and abnormal cell states (cluster 16 for mitochondrial highly expressed cells and cluster 13 that exhibits higher expression of stimulus-response genes) (Fig. 1b, Supplementary Fig. 6).

We recovered all major endosperm cell types at the early differentiation stage from the annotated cell clusters (Fig. 1b–f, Supplementary Fig. 8a). For example, canonical ESR marker genes, such as *Embryo-surrounding region (ESR)1*, *ESR2* and *ESR6*^20–22^, were more strongly expressed in cluster 15, suggesting that it corresponds to the ESR. We verified this assumption using ISH assays of cluster-15-representative genes *Maternally expressed gene* (*MEG*) *14* and *1d038758* (Fig. 1e, Supplementary Fig. 6). Clusters 2 and 9 were annotated as BETL based on differential expression of the marker genes *Myb related protein1* (*MRP1*), *BETL9*, *Transfer cell response regulator1* (*TCRR1*), and others^15,23–25^. We also verified this assignment by ISH of newly identified cluster-representative transcripts (*1d053108*, *1d052759* and *1d053785*), which both showed very strong signals at the bottom part of the endosperm. *Male sterile8* (*MS8*), *Sugars will eventually be exported transporter15a* (*SWEET15A*) and *Phosphatidylethanolamine-binding protein 11* (*PEBP11*)^9^, which are EAS cell markers, were more strongly expressed in cluster 11. Therefore, we assigned cluster 11 to the EAS. ISH analysis of *MS8* and *SWEET15A* indicated that cells highly expressed with these transcripts surround the upper region of the embryo at the stage of early differentiation, and are likely progenitors of the fully differentiated EAS (Fig. 1e, Supplementary Fig. 6). Of note, clusters 2, 9, 11 and 15 were clearly isolated on the Uniform Manifold Approximation and Projection (UMAP) plot, implying their transcriptome signatures are more distinctive (Fig. 1b). We designated clusters 1 and 8 as representing SE cells based on the expression of typical SE markers, including *Tryptophan aminotransferase related 1* (*TAR1*), *27-kDa zein protein* (*ZP27*) and *Shrunken2* (*SH2*)^26,27^, and on the ISH results observed for *Defective endosperm18* (*DE18*) and *Floury3* (*FL3*) transcripts^28^ (Fig. 1e, Supplementary Fig. 6). Based on the patterns of expression of the established AL markers *AL9* and *Vacuolar proton pump7* (*VPP7*)^29^, and the ISH results for cluster-representative transcripts *Subtilisin1* (*SBT1*) and *1d024210*, we assigned clusters 0, 3, 4 and 7 as representing the AL cell type (Fig. 1d, e, Supplementary Fig. 6). We annotated clusters 10 and 12 as proliferating cells because of the genes encoding mitosis phase markers, such as *HMG9* and other cyclin proteins^17–19^ were more strongly expressed (Fig. 1, Supplementary Figs. 2 and 6). Nevertheless, the ISH assay with *HMG9* indicated that this cell-cycle-related marker gene is broadly distributed across different cell types, including the embryo.

**Fig. 2:**
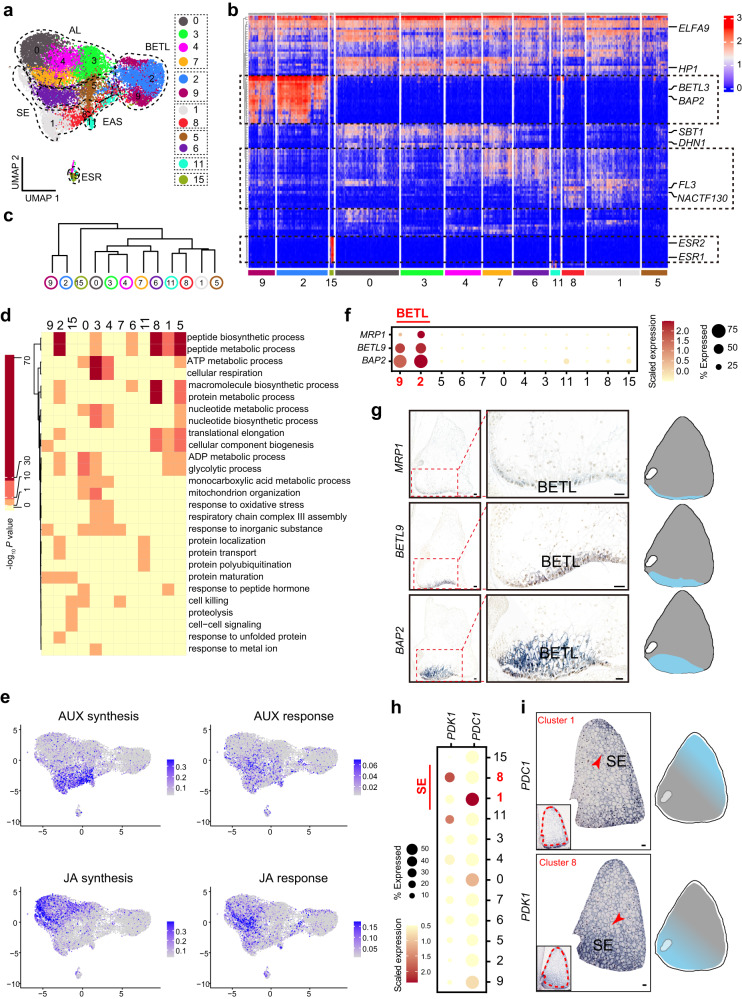
Endosperm cell types contain transcriptionally distinct subpopulations. **a** Visualization of five endosperm cell types by UMAP plots, colored according to cell clusters. **b** Heatmap depicting expression patterns of identified marker genes for major cell types. Selected classical marker genes and genes with known function are listed on the right. The colors from blue to red represent low to high expression levels. **c** Tree plot illustrating the relationship between clusters. **d** Representative GO terms enriched in different cell clusters. *P*-values were obtained using hypergeometric test, with g:SCS (graph-based stratified Cox-Snell) correction applied for multiple comparisons. **e** UMAP plots of gene-expression patterns related to auxin (AUX) and Jasmonic Acid (JA) biosynthesis and response. Colors ranging from gray to blue represent low to high expression levels. **f**, **h** Dot plots displaying selected markers of BETL and SE cells. **f** BETL, **h** SE. Circle size indicates the percentage of cells expressing the marker and color represents scaled expression values. **g**, **i** mRNA in situ hybridization results of genes shown in (**f**) and (**h**); scale bars, 100 μm. Experiments were repeated three times yielding similar results.

After analyzing the differentially expressed genes in clusters 5 and 6, we found that they were not obviously marked by any reported marker, making it challenging to assign them to any known cell type (Supplementary Fig. 6, Supplementary Data 4). Although the overall expression pattern of the cluster-representative genes of cluster 5 had a close relationship with the SE, whereas those of cluster 6 resembled the AL (Figs. 1 and 2; Supplementary Figs. 5b and 7), ISH results of selected clusters 5 and 6 markers did not conclusively support their AL-like or SE-like identities, respectively. Thus, we kept cell clusters 5 and 6 as undefined (Figs. 1 and 2, Supplementary Fig. 8). To confirm our cell-type assignment of each cluster, we compared our annotation with published cell-type-specific transcriptomes, the results showing that our cell-cluster identity was highly consistent with that from laser-capture microdissection (LCM) data^15^ (Supplementary Fig. 8c).

Overall, we assigned the endosperm cell types to the computationally generated cell clusters using known marker genes and ISH experiments, and also discovered a large number of new cluster marker genes (Supplementary Fig. 5b, Supplementary Data 4). These results are a resource that will provide insight into progressive development of endosperm cell identities.

### Distinct clusters exist within the known endosperm cell types

Our initial results revealed that anatomically defined maize endosperm cell types comprise distinct cell clusters, implying undescribed heterogeneity within these compartments (Figs. 1 and 2a). We then focused on the features of 12 cell clusters assigned to defined cell types (Fig. 2a). To illustrate the relationships between cell clusters, we assessed the expression patterns of the top cluster-representative genes. We found that whereas cell types of the AL and the SE exhibit distinctive signature genes (e.g., *SBT1 and DHN1* for AL, and *NAC130 and FL3* for SE), they also shared numerous similarities in terms of other cluster-representative genes, including *ELFA9* and *HP1*. This suggests a close relationship between the AL and the SE, and implies that they are not fully differentiated at 7 DAP (Fig. 2b). In contrast, the transcriptome signatures of ESR cluster 15 and BETL clusters 2 and 9 were highly distinguishable from other cell groups (Figs. 1 and 2b), in accordance with past observations that BETL and ESR cells differentiate earlier than other cell types. The BETL is recognizable shortly after cellularization is completed, with the ESR also starting to differentiate as early as 4 DAP^3,7^. Furthermore, in agreement with the morphological structure and spatial location of EAS and SE cells at early stage, the transcriptome signatures of cells from cluster 11 (EAS) were very similar to those in clusters 1 and 8 (SE) (Fig. 2b, c, Supplementary Fig. 8a). These results serve to refine the relationships between cell clusters and their corresponding cell types at the molecular level.

To characterize the function of each cell cluster, we examined Gene Ontology (GO) annotation for the cluster-representative gene sets, finding that not only different cell types, but also different cell clusters within the same cell type were enriched for different GO terms (Fig. 2d, Supplementary Fig. 9, Supplementary Data 5). For example, GO terms related to “cell-cell signaling” were only enriched in the ESR (cluster 15) across all cell types, which is partially due to the presence of CLAVATA3/ESR (CLE) peptide genes implicated in cell-to-cell communication^30^. The GO term “cellular respiration” was highly enriched in AL clusters 3 and 4 but not in other AL clusters (Fig. 2d, Supplementary Data 5), implying that these cells were active in energy metabolism. The BETL is implicated in nutrient transport, defense and signaling^31,32^. Interestingly, we obtained different GO terms in comparing the two BETL clusters. GO terms related to “protein transport” and “protein localization” were enriched in BETL cluster 2, while “cellular component biogenesis” and “response to inorganic substance” were enriched in BETL cluster 9 (Fig. 2d).

Phytohormones are important in endosperm development^33^. We plotted the expression of functional genes related to phytohormone biosynthesis and signaling pathways on UMAP plots. Employing the uniformly distributed gene *Ubiquitin1* (*UBI1*) as an internal control, we found that expression of phytohormone biosynthesis and response genes varied across different cell clusters (Fig. 2e, Supplementary Fig. 10a–j, Supplementary Data 6). For example, auxin (AUX) biosynthesis genes were more strongly expressed in SE cluster 8 and cluster 5, and jasmonic acid (JA) biosynthesis genes in AL clusters 0, 4 and 7. In contrast, the transcriptional signals of AUX- and JA-response genes were not more strongly expressed in the same cell clusters (Fig. 2e). Thus, differences exist between the cell clusters in terms of the functioning of phytohormone signaling pathways, which is largely consistent with observations in plant roots^34,35^. As for the phytohormone-related genes, transcription signals of additional functional gene sets were also differentially expressed in certain cell clusters within a cell type (Supplementary Fig. 10k, Supplementary Data 6). Taken together, these results imply that different cell types as well as the cell clusters within a cell type have distinct biological functions.

To further explore the heterogeneities in the canonical cell types, we identified additional differentially expressed transcripts from those cell clusters within the same cell type (Supplementary Fig. 11a–c, Supplementary Data 7). For example, in BETL cells, *Bax inhibitor1* (*BI1*) was preferentially expressed in cluster 9, whereas *gpm832*, a canonical BETL marker, was highly expressed in cluster 2 (Supplementary Fig. 11a). *Pyruvate decarboxylase1* (*PDC1*) was highly expressed in SE cluster 1, whereas *Pyruvate orthophosphate dikinase1* (*PDK1*) was preferentially expressed in SE cluster 8 (Fig. 2h, Supplementary Fig. 11b). Similarly, for the AL, preferentially expressed genes in each cluster (0, 3, 4 or 7) were identified (Supplementary Fig. 11c).

Spatial patterns of cluster-preferential genes within the BETL and SE provide further evidence for the division of BETL and SE into sub-cell-type clusters, respectively. The fully developed BETL comprises multiple layers of transfer cells^36,37^, and there is a developmental gradient along the basal-apical axis^38,39^. Both the computational expression patterns of BETL marker genes and the experimental ISH results conformed to this gradient pattern (Fig. 2f, g). Therefore, BETL cluster 2 likely represents the transfer cells at the outermost basal layer, where most canonical BETL markers are expressed, with cluster 9 representing transfer cell layers that are closer to the endosperm interior. SE cells differentiate from the inner cells formed after cellularization via periclinal divisions of subalurone cells from the outer regions of the endosperm^7,40^. We hypothesized that cluster-preferential genes within the SE cell type may also exhibit specific spatial distributions. ISH results show that these tested probes have less pronounced spatial patterns compared to those observed in BETL. For example, *PDC1 and 1d045392*, which are preferentially expressed in SE cluster 1, was observed to be more strongly expressed in the outer layer of SE cells adjacent to the AL. On the other hand, the cluster 8 preferentially expressed genes *PDK1* and *CNGT1*, were located towards the bottom of SE cells, near the BETL and embryo. These results provide a basis for further dissecting their developmental difference (Fig. 2h, i; Supplementary Fig. 11g, h). However, the ISH results of all tested cluster preferentially expressed makers within AL do not provide spatially distinctive evidence for the four putative AL clusters (Supplementary Fig. 11d–f).

To conclude, these results highlight the heterogeneity of endosperm composition at single-cell resolution, and demonstrate complex transcriptional signatures within maize endosperm cell types.

### Transcriptome dynamics at the transition stage during endosperm differentiation

**Fig. 3:**
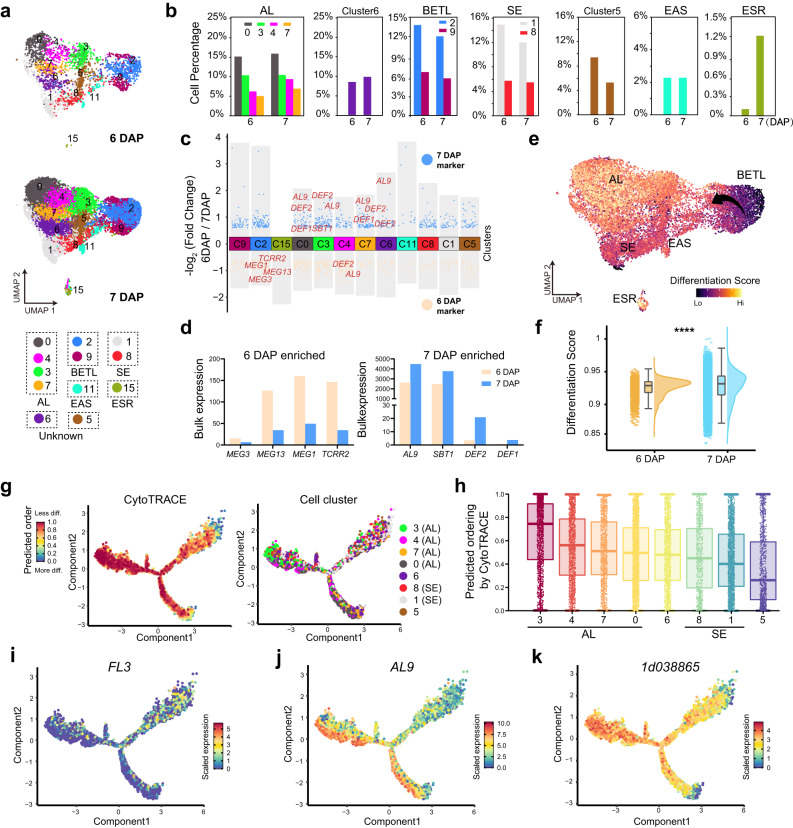
Transcriptome dynamics at the transition stage during endosperm differentiation. **a** Visualization of five endosperm cell types by UMAP plots at each time point, colored according to cell clusters. **b** Bar charts showing the ratio of cell number of different cell clusters at 6 and 7 DAP, colored as in (**a**). **c** Differential gene-expression analysis showing 6 DAP and 7 DAP marker genes across all 12 clusters. 6 DAP marker genes are indicated in milky white, while 7 DAP marker genes are indicated in blue. **d** Expression levels of selected marker genes at 6 and 7 DAP from bulk RNA-seq. milky white and blue represent genes with the highest expression at 6 DAP and 7 DAP, respectively. **e** Trajectories of the developmental “pseudotime” in each cell cluster mapped onto the same UMAP plot depicted in (**a**); the differentiation score is calculated based on the stage-specific genes identified from bulk RNA-seq. **f** Raincloud plot showing the cell differentiation score of 6 and 7 DAP. *n* = 2898 cells in 6 DAP, *n* = 12,698 in 7 DAP. ****, *P* < 0.0001. Two-tailed student’s *t*-test. No adjustments were made for multiple comparisons test. Box plots indicating median (middle line), 25th, 75th percentile (box) and 5th and 95th percentile (whiskers) as well as all data (single points). **g** tSNE plots of the SE and AL developmental trajectory depicting cell types (right) and pseudotime (left) using CytoTRACE. **h** Box plots showing CytoTRACE values for clusters 0, 1, 3, 4, 5, 6, 7 and 8. *n* = 2043 cells in cluster 0, *n* = 1644 cells in cluster 1, *n* = 1358 cells in cluster 3, *n* = 1216 cells in cluster 4, *n* = 732 cells in cluster 5, *n* = 1,161 cells in cluster 6, *n* = 968 cells in cluster 7. Box plots indicate median (middle line), 25th, 75th percentile (box) and 5th and 95th percentile (whiskers) as well as all data (single points). **i**–**k** Expression of selected genes in the pseudotime trajectory using CytoTRACE. **i** *FL3*; **j** *AL9*; **k** *1d038865*. Gradient from blue to red represents low to high expression levels.

Maize endosperm differentiation is arbitrarily separated into early (4–6 DAP) and late (8–12 DAP) stages^7^. To obtain molecular insight regarding the transition stage between early and late endosperm differentiation, we reanalyzed the transcriptomes of the 12 cell clusters at each time point. UMAP plots showed that all clusters were continuously detected from 6 and 7 DAP (Fig. 3a). However, the sizes of some cell clusters changed significantly across this time period (Fig. 3b). For example, the proportional cell number of ESR cluster 15 dramatically increased (from 0.1% at 6 DAP to over 1.2% at 7 DAP) (Fig. 3b). In addition, differential expression analysis revealed a cohort of genes with temporally specific enrichment in each cell cluster, indicating that these clusters were undergoing a vigorous differentiation process (Fig. 3c, Supplementary Data 8 and see Methods). We defined these as cell-cluster-time-specific (CCTS) genes. For example, expression of the defense related genes *Defensin-like protein* (*DEF*)*1* and *DEF2* of AL clusters 0, 3, 4, and 7 increased from 6 to 7 DAP (Fig. 3c, Supplementary Data 8). Interestingly, the temporal gene-expression patterns of each cell cluster corresponded to the cell-cluster identity, a large percentage of CCTS genes being also cluster-representative markers (Fig. 3c and Supplementary Data 8). For example, *Maternally expressed gene* (*MEG*)*1 and MEG3* were CCTS genes and also identified BETL cluster 2 markers (Fig. 3c). The dynamic expression patterns of selected CCTS genes were the same as the patterns observed in bulk RNA-seq data, further supporting a close relationship between temporal and spatial gene expression during early differentiation (Fig. 3d, Supplementary Data 8).

Next, we used the bulk RNA-seq gene-expression profiles of 6 and 7 DAP to identify temporally up- and downregulated gene sets (Supplementary Fig. 12, Supplementary Data 9 and see Methods). A cellular differentiation score for each cell was generated based on these gene sets, and finally resolved the developmental trajectories of cells in 12 clusters (Fig. 3e, f). The plot showed that the computationally defined cell clusters in BETL, which had functional heterogeneities, partially correlated with the differentiation state.

The AL and SE cell types are spatially adjacent and convertible under certain conditions^41–43^. To explore the common progenitor cells and cell fates required for their differentiation, we analyzed AL clusters 0, 3, 4, 7, SE clusters 1 and 8 and two adjacent clusters 5 and 6 from 7 DAP cells using CytoTRACE, a non-graph-based pseudotime prediction tool, which is able, without prior knowledge, to recover the direction of differentiation and identify immature cells. The Monocle2 software package projected the pseudotime-ordered cells into two distinct branches and revealed two terminal states representing more differentiated AL and SE (Fig. 3g). The cell ordering results indicated that AL cells were relatively less differentiated as compared to SE cells, as judged by the number of expressed genes (Fig. 3h). This result is also consistent with the cellular differentiation score analysis for each cell cluster (Supplementary Fig. 13). Surprisingly, besides cells exclusively marked by AL or SE markers (such as *AL9* for AL and *FL3* for SE), there were also cells having transcriptomic features derived from both cell types, which might imply they are their common progenitors (Fig. 3i–k, Supplementary Data 10). One gene that was highly expressed in these cells (*1d038865*) encodes a 60S ribosomal protein (Fig. 3k). Its Arabidopsis homolog, *AT2G19730*, is highly expressed in shoot apical meristem tissue and also marks the root quiescent center (QC) cells in a scRNA-seq analysis, suggesting meristematic commonalities for these predicted progenitor cells^44,45^. In addition, CytoTrace and “differentiation score” results were consistent with each other, further supporting the robustness of our analysis (Supplementary Fig. 13).

### Large-scale profiling of TF-binding sites using ampDAP-seq

**Fig. 4:**
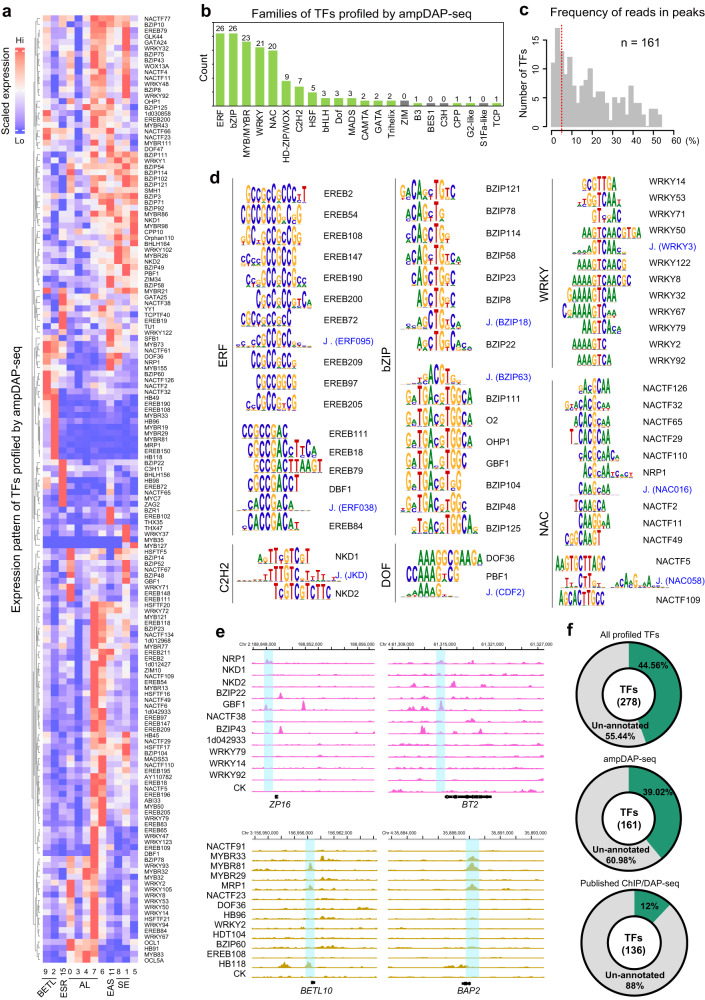
Genomic TF-binding sites profiling using ampDAP-seq. **a** Heatmap showing the average expression of the ampDAP-seq-tested TFs in each cell cluster. Gradient from blue to red represents low to high expression levels. **b** Summary of TF families profiled by ampDAP-seq. **c** Distribution of the fraction of reads in peaks (FRiP) score of each TF. **d** Clustering map of the representative motif identified for each selected TF. **e** IGV genomic track plots illustrating the targeting of genes by the selected SE or BETL-specific TFs. **f** Donut charts showing the fraction of the maize genome covered by TFBSs based on the published and ampDAP-seq data. The number of the associated unique TFs are indicated. The genome is colored in green according to the number of occupied TFs.

The transcriptomic identities of cell clusters are largely defined by their underlying gene regulatory networks (GRNs), in which TFs drive the expression of their target genes to establish distinctive expression profiles. Identifying the genome-wide TF-binding sites (TFBS) is essential for constructing GRNs containing direct targets of individual TFs. However, only a few genome-wide TFBS have been profiled in maize using chromatin immunoprecipitation sequencing (ChIP-seq) or DAP-seq^46–51^. We therefore tested over 200 TFs, selected based on their distinct cell-cluster expression patterns, to identify their genome-wide binding sites using optimized ampDAP-seq (Supplementary Fig. 14 and see Methods). Two rounds of ampDAP-seq enrichment increased the enrichment signal of stable DNA-binding events (Supplementary Fig. 14). We successfully recovered DNA-binding profiles and de novo predicted motifs for 161 TFs from 24 families (Fig. 4a–c, Supplementary Data 11).

Most of these motifs were consistent with those of the same TF family recorded in the JASPAR database, suggesting the high quality of our dataset (Fig. 4d, Supplementary Data 11). In total, we identified over 2 million (2,506,059) non-overlapping TFBS loci, with a median of about 79,258 binding sites per TF (Supplementary Data 11). Integrative Genomics Viewer (IGV) plots illustrated the genomic binding profiles of SE- and BETL-representative TFs at selected target gene sites, such as *16-kDa zein* protein (*ZP16*), *Brittle endosperm2* (*BT2*), *BETL10* and *Basal layer antifungal protein2* (*BAP2*), and recovered the binding peak of NRP1 and MRP1 to these known targets, further supporting the validity of our TFBS data (Fig. 4e). We also included additional genomic binding profiles (clustered into 311,832 non-overlapping loci) of 136 TFs acquired from published datasets in the following analysis^47,50,51^ (Supplementary Data 11). In total, genomic binding sites from 278 unique TFs resulted in an approximate 45% coverage in aggregate of the maize genome (Fig. 4f), which is more than the proportions of the genome associated with TF-bound and histone-modified regions found in the mouse (~13%) and human (~20%) genomes^52,53^. Of note, the proportion of TF-binding annotated regions may be underestimated since many regulators have not yet been tested. This result implies widespread regulatory potential in the maize genome.

### Identifying regulons using TF-binding profiles and the coexpression-based transcriptional regulatory networks

**Fig. 5:**
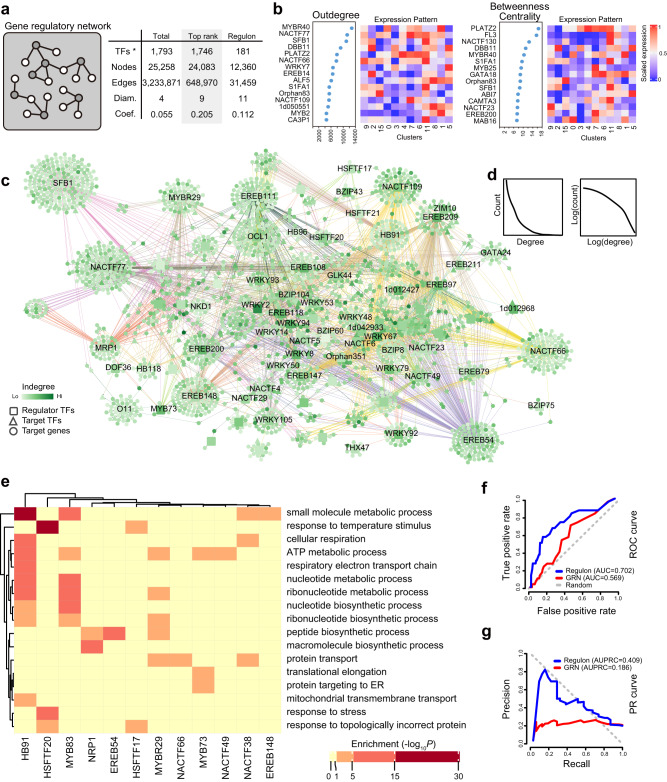
Reconstruction of a high-confidence maize transcriptional regulatory network. **a** Summary of the inferred GRN. Network topology parameters are shown. Diam., diameters; Coef., clustering coefficient; *, only regulatory TFs were counted. **b** Representative key regulators identified by network topology analysis (TFs with top out-degree and betweenness centrality are displayed); the expression patterns of these TFs are shown at right, with colors representing scaled expression values. **c** The regulatory network of 181 regulons. Cell-type-specific TFs are highlighted in the network. Colors represent the in-degree value determined by network topology analysis. **d** Distribution of node degree values fits the power law (black lines). **e** GO enrichments of selected regulons. Predicted target genes of representative TFs were enriched for functional terms associated with biological properties. *P*-values were obtained using hypergeometric test, with g:SCS (graph-based stratified Cox-Snell) correction applied for multiple comparisons. **f**, **g** ROC (**f**) and precision-recall (**g**) curves generated using known TF target genes for the coexpression GRN (red) and the integrated regulon GRN (blue).

The gene-expression profiles of individual cells provide an unprecedented opportunity to study the underlying gene regulatory programs^54^. We chose GRNBoost2, which is based on curated models, as our analysis pipeline. This yielded an interwoven network comprising 25,258 nodes (1793 regulator TFs) and 3,233,871 edges, covering almost all detected transcripts (Fig. 5a). Further filtering of this GRN to include only the top-ranked edges (see Methods), yielded more reliable connections from 24,083 nodes (1746 regulator TFs) (Fig. 5a, Supplementary Data 12). We identified potential key regulators based on network topology analysis (Fig. 5b, Supplementary Fig. 15, Supplementary Data 12). Well-known TF genes regulating endosperm development, such as *NAC130* and *FL3*^27,28^, possessed high “betweenness centrality” and “out degrees” scores, suggesting that our predicted network has high biological relevance (Fig. 5b, Supplementary Fig. 15).

After integrating the GRN and TFBS information from TFs profiled by ampDAP-seq, published DAP-seq and in vivo ChiP-seq in endosperm (Fig. 5a, Supplementary Fig. 16a), we obtained 181 regulons (TFs with their direct-binding targets) containing 12, 360 genes, having a median size of ~70 genes per regulon (Fig. 5c, Supplementary Fig. 16b, c, Supplementary Data 13). The in-degree and out-degree distribution patterns imply that these regulons form a scale-free network (Fig. 5d). We carried out GO enrichment analyses to link the regulons to functions in known biological processes. A set of regulons were indeed associated with specific biological functions (Fig. 5e, Supplementary Data 14). For example, the NRP1-regulated targets were associated with “peptide biosynthetic process” and macromolecule biosynthetic related GO terms (partially due to the corresponding zein protein encoding genes), which was consistent with their function in storage protein biosynthesis^27^.

Next, we evaluated the accuracy of predicted regulon targets using documented targets based on the RNA-seq and ChIP-seq analyses^15,46,49^ (Supplementary Data 15). Combining the coexpression GRN with TFBS significantly improved the predictive power of the network. Our coexpression GRN based on single-cell transcriptomes had an improved predictive power: the area under the Receiver Operating Characteristic (ROC) curve was 0.569, surpassing the 0.500 value for a random prediction. When we combined the TF-binding information to obtain a list of TF regulon targets, the area under the ROC curve (AUC) increased to 0.702 (Fig. 5f). Likewise, more true positive targets were recalled from the regulon-based network compared to the coexpression GRN for any given network precision setting (Fig. 5g). For instance, we recovered the reported targets of MRP1 (*TCRR1, MN1, MEG3*, *BETL10*, and others^15^) in our network (Supplementary Fig. 16d). To further verify our predicted network, we also examined the newly predicted targets of selected TFs by in vitro transactivation assays. Randomly selected newly identified targets from the regulons, such as EREB108, MYBR81 and WRKY8, were verified (Supplementary Fig. 16e). These results confirm successful reconstitution of a high-confidence endosperm transcriptional regulatory network.

### Identifying essential regulators of cell-cluster identity

**Fig. 6:**
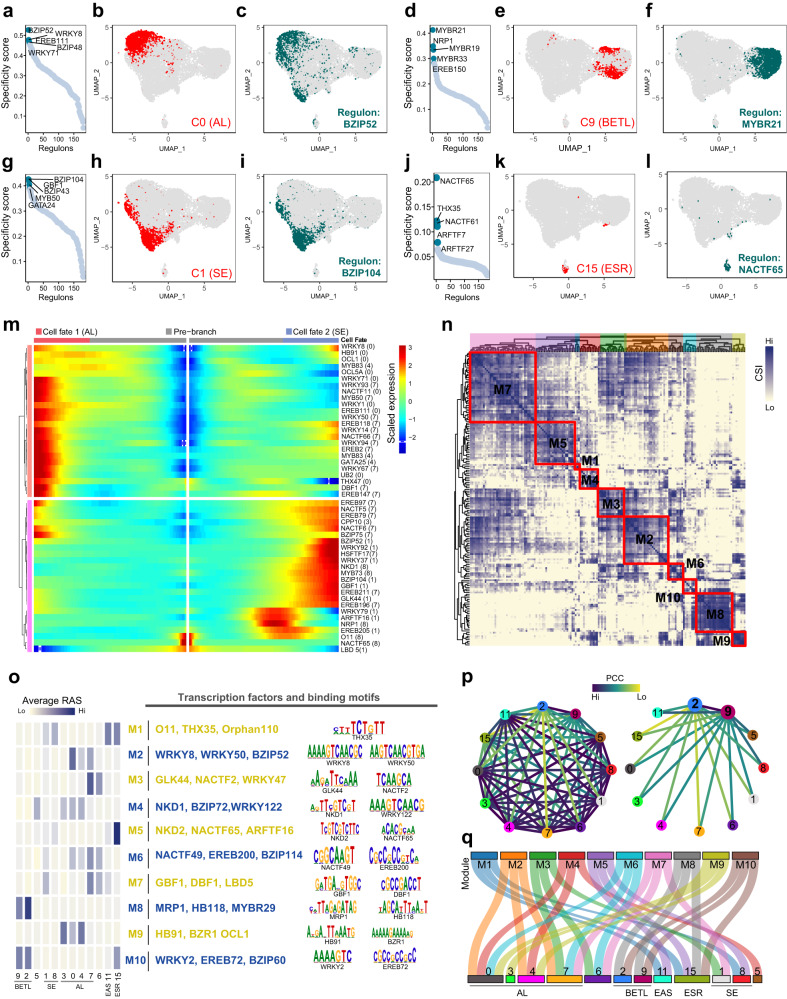
Regulons form modules and coordinate their activity within cell types. **a** Ranking of regulons in cluster 0 based on the regulon specificity score (RSS). **b** Cluster 0 cells are highlighted in the UMAP plot (red dots). **c** Binarized regulon activity scores (RAS) (Z score normalized across all samples, and 0.15 is set as the cutoff to convert to 0 and 1) for a regulon (BZIP52) with the top RSS value on UMAP plot (dark green dots). **d**–**f** Same as (**a**–**c**) but for cluster 9 and regulon MYBR21; 0.079 set as the RAS cutoff. **g**–**i** Same as (**a**–**c**) but for cluster 1 and regulon BZIP104; 0.004 set as the RAS cutoff. **j**–**l** Same as (**a**–**c**) but for cluster 11 and regulon NACTF65; 0.048 set as the RAS cutoff. **m** Heatmap showing gene-expression pattern during differentiation of AL (fate1), SE (fate2) along pseudotime. **n** Identified regulon modules along with selected TFs and corresponding binding motifs. **o** Heatmap showing average activity scores of 10 modules in different cell clusters. **p** Network of relatedness for the selected 12 cell clusters constructed based on similarities between regulon activities. **q** Sankey plot displaying relationships between cell cluster and the top 3 regulons in modules M1–M10.

To link the regulatory functions predicted by the GRN to individual cell clusters, we searched for the critical regulators for cell identity by calculating the regulon specificity score (RSS) for the cell clusters based on Jensen-Shannon divergence and regulon activity scores (RAS) using the AUCell algorithm (Supplementary Data 16 and 17), and identified the most specific regulons (having the highest RSS values) associated with each cell cluster (Fig. 6a–l, Supplementary Fig. 17). In agreement with previous studies, MRP1, a master regulator of BETL development^24^, was one of the most specific regulons associated with BETL cluster 9. Our network analysis also identified O11 and NKD2 as two of the most specific regulons associated with SE cluster 8; both are well-known regulators of SE^46,55^. The UMAP plot, including the binarization score for each cellular regulon activity, supported the idea that the activities of these regulons were highly specific to the indicated cell clusters (Fig. 6a–l, Supplementary Figs. 17 and 18). These results suggested a successful recapitulation of critical regulators for defined cell clusters using this approach. In addition, we identified a new list of essential TFs for each cell cluster. For example, BZIP52, WRKY8, EREB111, BZIP48, and WRKY71 had the highest RSS values associated with the AL cluster 0 (Fig. 6a); NACTF65, THX35, NACTF61, ARFTF7, and ARFTF27 had the highest RSS values associated with the ESR cluster 15 (Fig. 6j). To establish a connection between the regulons and the differentiation dynamics of AL and SE, we conducted embedded heatmap analyses of the regulator TFs that belonged to the most specific regulons in clusters 0, 3, 4, 7, 5, 1, 8 and 6. Our analysis revealed that the TFs linked with the differentiation of these cell types exhibit unique temporal expression patterns along pseudotimes without any overlap (Fig. 6m). In conclusion, our findings provide a valuable resource for identifying key regulators of endosperm tissue differentiation, although the specific functions of these newly predicted regulators in each cell cluster require further investigation.

### Regulons involved in endosperm differentiation form combinatorial modules

To analyze the relationships between the cell-cluster-associated regulators, we systematically characterized the combinatorial patterns exhibited by the TFs and their targets. We compared the regulon activity ratings of each pair of regulatory relationships using the connection specificity index (CSI). After filtering out the low-quality regulons and those with less than five targets, we classified 168 regulons into 10 modules (M1–M10) (Fig. 6n, Supplementary Fig. 19a, Supplementary Data 18–21 and see Methods). Through mapping the average activity score of each module onto the cell-identity UMAP map, we found that each module occupies a distinct region, showing not only cell type but also cell-cluster preferences (Supplementary Fig. 19b). We also linked the modules, which included unique regulators and their binding motifs, to cell clusters based on their average activity scores (Fig. 6o). For example, module M8 is enriched in clusters 2 and 9, and contained MRP1^24^, an essential regulator for the BETL; module M1 is enriched in clusters 1 and 8, it contained O11^46^, a central hub of maize endosperm development and of the regulatory network of zein protein synthesis (Fig. 6o).

Related cell clusters of a given cell type shared similar network structures, as depicted in a highly-modularized graph in which two related cell clusters with similar total regulon activities are connected by an edge having a higher Pearson correlation coefficient (PCC) value. The higher the PCC value between cell clusters, the more likely they are to share the same regulons (Fig. 6p, Supplementary Data 22). For instance, the PCC value between cell clusters 2 and 9 of BETL is higher than those between cluster 2/9 and other cell clusters, suggesting a stronger correlation between them. This result further confirmed the accuracy of our cell clustering from the perspective of regulatory modules.

Further, when we used Sankey diagram to highlight the link between the cell clusters and the regulon modules, we found that the same cell cluster corresponded to multiple modules. (Fig. 6q). For example, M2, M4, and M9 were linked to cluster 0 belonged to AL cells, and M8 and M10 were correlated with cluster 2, representing BETL cells. Thus, we propose that the developmental fate of a cell cluster may be regulated by multiple core regulatory networks.

**Fig. 7:**
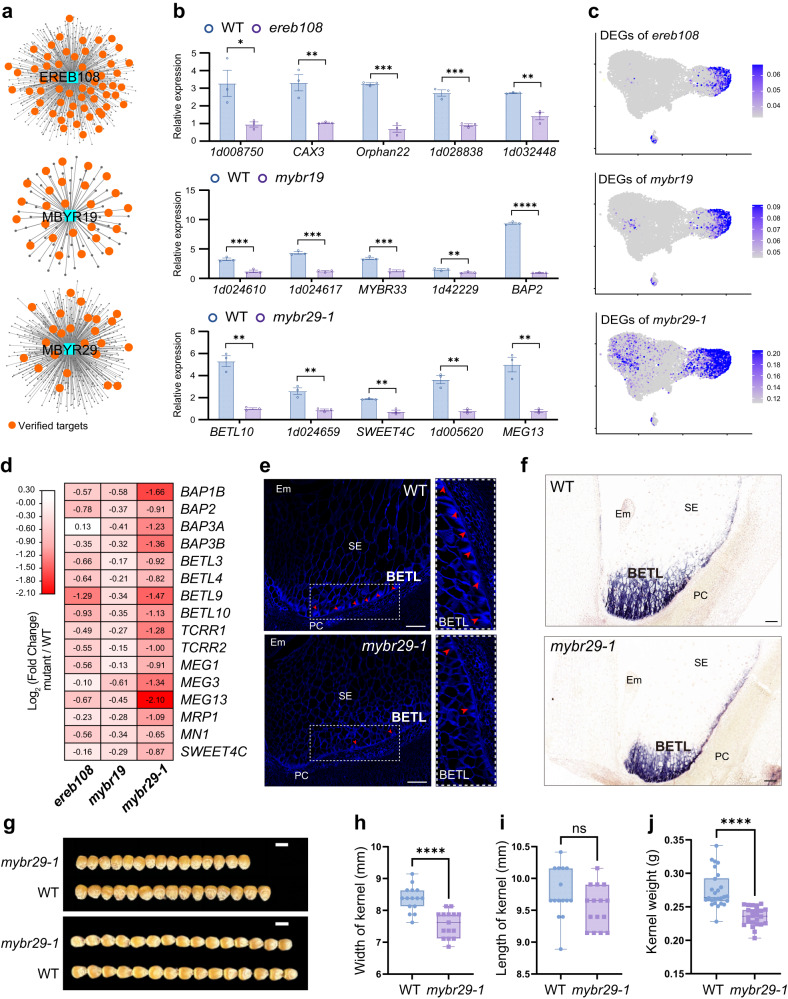
Validation of important regulons in BETL cell type. **a** The gene regulatory network regulated by three transcription factors. The gray color represents the target genes in the regulon, while the orange color indicates differential expression of these genes in the corresponding mutants. **b** Transcript levels of target genes in three regulons in WT and mutants. Error bars indicate ± SEM (*n* = 3 biologically independent samples). *, *P* < 0.05; **, *P* < 0.01; ***, *P* < 0.001; ****, *P* < 0.0001. Two-tailed student’s *t*-test. No adjustments were made for multiple comparisons test. **c** UMAP plots of gene-expression patterns related DEGs of three mutants. The colors from gray to blue represent low to high expression levels. **d** The heatmap illustrates the log_2_ (fold change) of selectively genes that are predominantly expressed in BETL and detected in three mutants endosperms. The colors from white to red represent low to high fold change. **e** Images of endosperm stained with calcofluor white between WT and *mybr29-1*. CWIs appear light blue with calcofluor white staining (indicated by red arrowheads); scale bars, 50 μm. Experiments were repeated three times yielding similar results. **f** mRNA in situ hybridization results of *1d053785* between WT and *mybr29-1*; scale bars, 100 μm. **g** Phenotypic features of the *mybr29-1* mutant. Scale bars, 1 cm. **h**, **i** Kernel length and width of WT and *mybr29-1* mature kernels. *n* = 15 kernels in WT, *n* = 15 kernels in *mybr29-1* mutant. ****, *P* < 0.0001. Two-tailed student’s *t*-test. No adjustments were made for multiple comparisons test. Box plots indicate median (middle line), 25th, 75th percentile (box), and 5th and 95th percentile (whiskers) as well as all data (single points). **j** Kernel weight of WT and *mybr29-1* mature kernels. *n* = 26 kernels in WT, *n* = 24 kernels in *mybr29-1* mutant. ****, *P* < 0.0001. Two-tailed student’s *t*-test. No adjustments were made for multiple comparisons test. Box plots indicate median (middle line), 25th, 75th percentile (box) and 5th and 95th percentile (whiskers) as well as all data (single points).

In our work, we further validated a number of novel functional regulons predicted from the network analysis. For example, we identified EREB108, MYBR19 and MYBR29 as key regulators associated with BETL clusters 2 and/or 9, implying their crucial roles in BETL development (Fig. 6, Supplementary Figs. 17 and 20). To confirm their predicted targets, we conducted RNA-seq experiments on homozygous mutants of these TFs at 8 DAP (Supplementary Fig. 20a–c). The results showed that the predicted targets of these TFs were well-supported by the differentially expressed genes (DEGs) in their mutants (Fig. 7a, b and Supplementary Fig. 20b, Supplementary Data 23–25). Moreover, the DEGs of these mutants exhibited higher average expression levels in BETL (Fig. 7c). Previously reported BETL key genes, such as *MRP1* and *SWEET4C*^56^, were downregulated in these mutants, suggesting defects in BETL differentiation (Fig. 7d). Microscopic examinations revealed that the transfer tissue in *mybr29* mutants comprises cells lacking or having fewer cell wall ingrowths (CWI) compared with wild type (Fig. 7e). Consistent with this histological observation, the ISH test of *1d053785*, a BETL marker gene, showed a lower abundant and a more restricted region of expression in the mutant endosperm (Fig. 7f, Supplementary Fig. 20e). A small-seed with decreased kernel weight phenotype in mature seeds of *mybr29* mutants compare with wild type were also observed and probably due to these differentiation defects in BETL (Fig. 7g–j, Supplementary Fig. 20f–h). Collectively, these results further support the important roles of MYBR29 in BETL development. In summary, the transcriptome signature and developmental defects in these loss-of-function mutants indicated that these TFs are key regulators for BETL development.

## Discussion

Improvement of grain yield and quality in cereal crops requires better understanding of the regulation of cereal endosperm development. Single-cell transcriptomes have increased analytical resolution to the level of an individual cell^57^, with two single-nucleus RNA profiling studies on early developing syncytial endosperm tissues reported in *Arabidopsis*^58,59^. Although the persistent cereal endosperm is similar to that of *Arabidopsis* in early development (from coenocytic nuclear divisions to cellularization to form cell walls between individual nuclei), the cells later differentiate into additional cell types and rapidly accumulate storage products^4^. Our scRNA-seq analysis and regulatory network inference of the maize endosperm, which develops more cell types than other cereal crop species, provides the most detailed information to date of transcriptional regulation in early endosperm development.

Here, we have constructed a comprehensive transcriptome compendium and underlying GRN of the maize endosperm during the differentiation stage, unveiling a previously unrecognized complexity. For example, our analysis indicated that both BETL and SE cell types comprise two cell clusters, respectively (Fig. 2). Positioning is important for AL, which is the epidermal layer covering most all the endosperm surface, cell specification, as determined through in vitro culture of isolated endosperm^42,60^. The AL cells adjacent to the embryo, defined as the scutellar AL, are characterized by strong expression of putative transporter-encoding genes^9^. These findings suggest that distinct positional cues lead to the characteristics of AL cells during formation. Despite having four computational cell clusters for AL, the ISH results of selected markers did not support their distinct spatial expression, suggesting the differential gene expression detected in these cell clusters may not solely be due to the different positional distribution of AL cells (Supplementary Fig. 11).

We note that BIZ and CZ are two cell types uncaptured in our study, possibly because they differentiate later in endosperm development^7,61^. CZ cells are highly elongated, with large nuclei and sparse cytoplasm^7^, and are not apparent at the early differentiation stage. The BIZ comprises 2–4 layers of cells that are located internally to the BETL and not fully established by 8 DAP^7^. However, we detected that *BURP9*, a marker exclusively localized in the BIZ^12^, as being more strongly expressed in cluster 9. Combined with the observation of a gradient expression pattern of other BETL marker genes, some of the cells in cluster 9 may later differentiate into BIZ cells (Fig. 2f, g).

In scRNA-seq data, the captured cells can often originate from different cell-cycle stages. We attempted to remove cell-cycle effects on clustering from the analysis. To do so, we have collected a number of reported maize cell cycle marker genes and homologs based on a study in rice. However, a small group of cells are still annotated as being in the G2/M phase (Fig. 1b), which could be due to the incomplete annotation of cell cycle marker genes.

The rapid growth of scRNA-seq datasets has opened new opportunities for coexpression-based GRN prediction. Compared to transcriptomes obtained from bulk RNA-seq experiments, one scRNA-seq experiment can generate a large number of samples, which allows capture of additional meaningful biological variation^62^. Although earlier maize GRNs based on bulk transcriptomes have been constructed for roots, stems, leaves, and seeds^14,63,64^, they all lack of TF-binding site information. In our work, we have constructed a transcriptional regulatory network of maize endosperm during cell differentiation that combines a coexpression-based GRN with newly characterized TF-binding profiles. These results greatly expand understanding of the complex endosperm regulation system, and imply that plants, like animals, have complex and redundant regulatory relationships, which might sustain adaptation to the environment and influence evolutionary processes^51,65–67^.

Because cell identity is determined by the activity of TFs, cell-type-specific regulons are important for plant cell differentiation and are targets for crop breeding^68^. We have identified cell-type-specific regulons for different endosperm cell types and dissected their regulatory relationships and biological functions. As an illustration, we confirmed the roles of three essential regulator TFs in the BETL (Fig. 7). Furthermore, the target genes of AL specific regulon HB91 were annotated as “response to inorganic substance”, suggesting they may play an important role in mineral accumulation. As AL cells are known to be important for mineral storage^69,70^, *HB91* and its associated target genes are of particular interest for further analysis in this area.

TFs in plants often act in concert to coordinate gene expression^71^. Regulons in our maize endosperm GRN also coordinately regulate cell type development (Fig. 5). Regulon combinations can form thousands of regulatory relationships, and their modularity also facilitates identification of the mechanisms of gene mutation^72^. Although the regulons we have identified are in endosperm cells, this approach can also be readily applied to other maize tissues. Comprehensive analysis of regulons in all maize tissues using the same strategy should greatly expand the repertoire of regulons and our understanding of the regulon-based regulatory network in this important crop.

Finally, we have made all our data publicly available online (https://www.maize-endosperm.cn), providing a web interface to enable researchers to easily navigate the expression and regulatory network atlas. Our regulatory atlas of the endosperm covers cell clusters/types, and TFs and their targets from early to late differentiation. The web interface provides gene-expression-centric and regulon-centric views. As such, we have created a valuable resource for the broad biological community.

## Methods

### Maize endosperm protoplast isolation

Maize (*Zea mays*) inbred line B73 endosperm protoplasts were isolated as previously described with some modifications^73^. Briefly, 80–100 maize kernels at 6 and 7 DAP were harvested. The endosperms were rapidly separated using a sharp surgical blade and placed on Murashige and Skoog (MS) agar medium, then quickly cut and transferred to a 90 mm Petri dish containing 20 mL of freshly prepared enzyme solution. The enzyme solution contained 1% (w/v) cellulase R-10 (Yakult Pharmaceutical), 0.75% (w/v) macerozyme R-10 (Yakult Pharmaceutical), 0.1% Pectolyase Y-23 (Yakult Pharmaceutical), 0.4 M mannitol, 20 mM KCl and 20 mM MES (pH 5.7). The enzyme solutions were incubated in a 55 °C water bath for 10 min. After being cooled to room temperature, 10 mM CaCl_2_, 0.1% (w/v) BSA and 0.035% (v/v) 2-mercaptoethanol were added and then the entire enzyme solution was filtered through a 0.22 μm filter. The endosperms were digested in the dark at room temperature for 2 h with agitation at 10 rotations per min. Then, an equal volume of releasing buffer (150 mM NaCl, 125 mM CaCl_2_, 5 mM KCl and 20 mM MES) was added to the enzyme mixture and gently shaken for 15 s to release protoplasts. The protoplast-enzyme suspension was filtered through a 70 µm cell strainer and the filtrate was gently horizontally centrifuged at 110 × *g* for 3 min to pellet the protoplasts. The protoplasts were washed twice in 20 ml washing solution (0.5 M mannitol, 10 mM CaCl_2_, 20 mM KCl and 20 mM MES) and filtered twice through a 40 µm cell strainer, then brought to a concentration of 600–1000 protoplasts/µL. Protoplast viability was determined by the trypan blue staining method.

### Single-cell RNA sequencing

The scRNA-seq libraries were generated using the Chromium Single Cell 3′ Reagent v3 Kit with the 10× Genomics protocol. Briefly, protoplasts were loaded onto the chromium chip; then, the Chromium Single Cell Controller Instrument was used to generate single-cell gel beads in emulsions (GEMs). Incubation of the GEMs produced barcoded, full-length cDNA. The GEMs were then broken, and the barcoded cDNAs were cleaned up using Silane magnetic beads and amplified via PCR. After fragmentation, end-repaired and A-tailing, index adapters were ligated, and barcoded cDNA libraries were constructed. The amplified libraries were sequenced on the NovaSeq platform (Illumina) to by Berry Genomics (Beijing) generate 150-bp paired-end reads, according to the manufacturer’s instructions.

### DNA affinity purification sequencing

The ampDAP-seq experiment was performed as previously described with modification^74^. Briefly, genomic DNA (gDNA) was extracted from the leaves of the maize inbred line B73. PCR-amplified gDNA libraries were prepared using the TIANSeq DirectFast Library Kit (cat. no. NG101, TIANGEN) according to the manufacturer’s instructions. Full-length maize TF coding sequences were cloned from maize endosperm cDNA into the NotI and AscI sites of pUC57-Halo using MultlF Seamless Assembly Mix (cat. no. RM20523, ABclonal). TFs were synthesized using TNT SP6 High-YIELD Wheat Germ Master Protein Expression System (cat. no. L3260; Promega) according to the manufacturer’s instructions. Halo-fusion protein was bound to Magne HaloTag Beads (cat. no. G7281; Promega) and washed three times using TBSN buffer (100 mM Tris buffer, pH 7.5, 50 mM NaCl and 0.005% NP-40). Then, 500 ng gDNA libraries was added and incubated at room temperature for 1.5 h. After incubation, the beads were washed four times. Beads were resuspended in 30 µl of EB buffer to recover DNA. The recovered DNA was cleaned up with Silane magnetic beads and amplified via PCR. Two successive rounds of affinity purification were performed. The final PCR products were cleaned with Silane magnetic beads and sequenced on the NovaSeq platform (Illumina) by Berry Genomics (Beijing) to produce 150-bp paired-end reads.

### Bulk RNA-seq

Twenty maize B73 endosperms obtained at 6 and 7 DAP were pooled to extract total RNA with an RNAprep Pure Plant Kit (cat. no. DP441, TIANGEN), respectively. Three biological replicates were made from the kernels of three independent ears of maize. cDNA libraries were constructed following Illumina standard protocols and sequenced on the NovaSeq platform (Illumina) by the Berry Genomics company (Beijing) to produce 150-bp paired-end reads.

### mRNA in situ hybridization

mRNA ISH was performed as described previously with some modifications^56^. Briefly, sequences of marker-gene cDNAs were used to design the probes. All probes were synthesized and labeled using the DIG RNA Labeling Kit (SP6/T7) (cat. no. 11175025910, Roche) according to the manufacturer’s instructions. 7 DAP kernels of the B73 inbred line were formalin-fixed and paraffin-embedded for sectioning. 8 μm sections were hybridized with the labeled probes, reacted with anti-digoxigenin-alkaline phosphatase (AP) (cat. no. 11093274910, Roche), and detected using nitro-blue tetrazolium chloride (NBT) and 5-bromo-4-chloro-3′-indolyphosphate p-toluidine salt (BCIP) stock solution (cat. no. 11681451001, Roche). Relevant primer sequences are given in Supplementary Data 26.

### Dual-luciferase transient transcriptional activity assay

The dual-luciferase transient transcriptional activity assay was performed as previously described^75^. To generate promoter reporters for the assays, the 1000-bp promoter fragment upstream of the transcription start site (TSS) of the genes were inserted at the Kpn1 and HindIII sites of pGreenII 0800-LUC using MultlF Seamless Assembly Mix (cat. no. RM20523, ABclonal). 35 S:TF effectors were created by inserting their coding sequences into the HindIII and XbaI site of the pHB vector using the MultlF Seamless Assembly Mix. The empty vector was used as a negative control. We performed transient dual-luciferase assays in mesophyll protoplasts of maize B73 etiolated leaves. Firefly luciferase (LUC) and Renilla luciferase (REN) activities were measured with dual-luciferase assay reagents (cat. no. E1960; Promega) using a microplate reader (SPARK, TECAN). We calculated the ratio between LUC and REN activities with at least three biological replicates. Relevant primer sequences are given in Supplementary Data 26.

### ampDAP-seq analysis

Trimmomatic (version 0.39)^76^ was used to trim the fastq files as follows: ILLUMINACLIP: TruSeq3-PE-2.fa:2:30:10, LEADING:3, TRAILING:3, SLIDINGWINDOW:4:15, HEADCROP:0, MINLEN:75; and bowtie2 (version: 2.4.1)^77^ was used to align them to the B73 v4 reference genome with the following parameters: -I 75 -X 1000 --no-discordant --no-mixed. The “MarkDuplicates” tool of Picard (version 2.25.6) was used to remove the duplicates formed by PCR amplification. Peaks were identified using GEM (version 3.4)^78^ using a background subtraction sample of the pUC57-Halo control sample. Peak identification was identified using the following parameters: --k_min 4 --k_max 12 --fold 3 --q 3 --nrf --outNP --outHOMER –outMEME. Individual datasets were transformed into bigWig format using bamCoverage from deepTools (version 3.5.1) with a bin size of 100 bp and normalized by counts per million (CPM) for visualization. Integrative Genomics Viewer (IGV) (version 2.10.0) was used to visualize the bigWig files. Peak annotation was performed with the B73 v4 reference genome annotation file created by maize TxDb based on GenomicFeatures (version 1.44.0)^79^, and then ChIPseeker (version 1.28.3)^80^ was used to determine peak annotation. The promoter region was defined as the 3000 bp region upstream and downstream of the TSS. To identify target genes for the regulons, high-confidence target genes were defined as those having a peak within 1 kb upstream and 1 kb downstream of the TSS.

### Analyzing the detection and enrichment of transcription factor binding sites

First, the top 600 peaks were sorted based on their *q*-value and fold enrichment. Then, the 200 bp sequence located at the summit of each peak was analyzed for enriched motifs using two methods. De novo motifs were identified using MEME-ChIP from the MEME software toolkit (version 5.5.1)^81^, whereas known motifs from the JASPAR 2022 database were detected using TOMTOM^82^ and Centrimo^83^ from the same toolkit.

### Bulk RNA-seq analysis

Trimmomatic (version 0.39)^76^ was used to trim the sequence reads, and STAR (version: 2.7.5c)^84^ was used to align them to the B73 v4 reference genome. Gene-expression levels for uniquely mapped reads were computed with featureCounts (version 2.0.1)^85^. To conduct a correlation study of gene expression comparing endosperm tissues before and after protoplast preparation, as well as between the bulk RNA-seq and the integrated scRNA-seq data, the log_2_(mean CPM+1) expression levels for each gene were computed and the Pearson correlation coefficient (PCC) was calculated in R.

### Cell-type clustering and marker-gene identification

The scRNA-seq data at 6 and 7 DAP samples were aligned and counted using the Cell Ranger pipeline (version 4.0). For additional data analysis, the raw count matrix was imported into R using the Seurat (version 4.0.3) package^86^. Cells with less than 500 but more than 7500 features were filtered, as were cells with fewer than 1000 counts and cells with a log_2_(feature/count) of less than 0.8. The data was then normalized using the “NormalizeData” function with the LogNormalize method and a scaling factor of 10,000. We identified variable genes using the “FindVariableGenes” function with the vst method and selected 2000 features. The data was then scaled using the “ScaleData” function. We performed principal component analysis (PCA) using the “RunPCA” function and retained 50 principal components. The significance of the PCA scores was determined using the “JackStraw” function, and all datasets were integrated using Harmony^87^, clustered according to resolution = 0.75, and the clustering binning results visualized using the Seurat functions RunUMAP and RunTSNE (dim = 1:50), respectively. In order to address the impact of cell cycle heterogeneity on cell clustering, the “CellCycleScoring” function was utilized to determine the cell cycle score for each individual cell, based on the cycling orthologous genes found in rice and maize known cycling gene (Supplementary Data 3). The “ScaleData” function was then employed to eliminate these cell cycle effects, utilizing the “vars.to.regress” parameter. When selected clusters were extracted to generate plots, the data was re-normalized and re-scaled prior to the downstream analyses. The function FindAllMarkers (Wilcoxon rank-sum test, min.pct = 0.3, logfc.threshold = 0.25) was used to find all the marker genes in each cluster. Temporal differential expression for each cell type was analyzed using the Seurat package’s FindAllMarkers(Wilcoxon rank-sum test, min.pct = 0.3, logfc.threshold = 0.25) function, treating each cell as a replicate and applying Bonferroni correction for p-value adjustment.

### Doublet detection

DoubletFinder algorithm (version 2.0.3)^88^ was employed to identify potential doublets by utilizing three input parameters: expected real doublets (nExp) calculated as the cell count divided by 100,000, artificial doublets (pN) set at 0.25, and neighborhood size (pK). To infer the optimal pK value, paramSweep_v3, summarizeSweep, and find.pK functions were used for a more comprehensive analysis.

### Identification of one-to-one orthologs

To identify homologous genes between maize and rice, we downloaded the maize and rice immediate homologous gene correspondence table from maizeGDB (https://download.maizegdb.org/Zm-B73-REFERENCE-GRAMENE-4.0/Orthologs/ZmB73v4.Gramene_53.rice_orthologs.txt), and the correspondence was divided into one-to-one immediate homologs, one-to-many groups, and many-to-many groups, and we used only one-to-one immediate homologs for the conversion.

### Gene ontology enrichment analysis

GO enrichment analysis was performed with gprofiler2 (version 0.2.0)^89^ in R. The default hypergeometric test was employed for determining statistical significance. The g:SCS (graph-based stratified Cox-Snell) algorithm was used for multiple testing correction.

### Pseudotime analysis

The Monocle2 (version 0.2.1)^90^ and CytoTRACE (version 0.3.3)^91^ programs were used to analyze cell differentiation throughout pseudotime and determine the destiny of individual cells. An analysis was performed on a portion of the raw data with target clusters to investigate the developmental trajectory of particular cell types. The specific steps were as follows: First, the dispersiontable () function was used to determine the variation across cells in the expression of each gene. The highly variable genes were ordered according to their average expression. Second, we used the reduceDimension function (set max components = 2, method = ‘DDRTree’) to reduce the dimensionality. The cells were finally placed in order using the orderCells () function. Plot cell trajectory in Monocle2 was used to create the trajectory. The differentiation potential of the cells was investigated using CytoTRACE; all settings were left at their default values. CytoTRACE scores range from 0 to 1, with lower values indicating more differentiation and higher values indicating less differentiation.

### Cellular differentiation score

Bulk RNA-seq data from endosperm of 6 and 7 DAP at two different stages of endosperm development were analyzed using edgeR (version 3.34.0)^92^. The significantly expressed genes were identified based on the following criteria: a FDR (False Discovery Rate) of less than 0.05 and a log_2_-fold change greater than 2, with endosperm from the 6 DAP period serving as the control group. The gene signature scores (GSCs) of 6 and 7 DAP marker genes were calculated using UCell (version 1.0.0)^93^. The cellular differentiation score was calculated as a log_2_ ratio of all 7 DAP/6 DAP marker genes GSCs.

### Identifying regulons and inferring their activity

GRNboost2 (version 0.1.6)^94^ was used to generate GRNs from scRNA-seq data. The single-cell expression matrix and TF list were used to identify coexpression modules between TFs and probable target genes, averaging 40× results to obtain reliable coexpression modules. The top 1 million regulatory pairs were extracted to form the top-ranked GRN. TF direct target genes for each coexpression module were inferred from the high-confidence target genes in the ampDAP-seq, DAP-seq and ChIP-seq of the associated TF. Next, each regulon was defined as a collection of TFs and their direct target genes. The result was visualized by Cytoscape (version 3.8.0)^95^. AUCell (version 1.14.0)^96^ was used to identify cells with an active regulon in single-cell RNA-seq data. The regulon active scores (RAS) in each individual cell were determined by the area under the recovery curve. In order to calculate the RAS, we separately checked whether the genes in the regulon were within the top 1%, top 5%, top 10%, top 15% and top 20%, and then determined the most appropriate parameters for each regulon (Supplementary Data 21). The RAS estimates the proportion of genes in the regulon that are highly expressed in each cell. The function “AUCell_exploreThresholds()” was used to automatically plot all histograms and calculate several thresholds that can be used to consider regulon “activity”, using the default threshold.

### Defining regulon specificity scores

A previously published entropy-based approach was used to calculate regulon specificity scores (RSS) to assess a regulon’s cell-type specificity^97^ in R with the package philentropy (version 0.5.0). The RAS matrix was used as the input matrix to calculate RSS. The top 5 most specific regulons were displayed using the R package ggplot2 (version 3.3.5).

### Regulon module analysis

The connection specificity Index (CSI)^98^ was used to discover regulon modules. The CSI is a context-dependent metric for detecting particular associated partners. The assessment of CSI is a two-step process. Firstly, the PCC of activity ratings for each pair of regulons is determined. The CSI of regulon P and regulon Q is defined as the proportion of all regulon pairs correlated with P, Q for which the PCC is less than PCC(P, Q). The larger the CSI, the greater the correlation between regulon A and regulon B.

The CSI of regulons P and Q is calculated as follows:

$${m}_{i}^{P,Q}=	 \left\{\begin{array}{cc}1 & {{{\rm{PCC}}}}\left(P,i\right) \, < \, {{{\rm{PCC}}}}(P,Q)\,{{\mbox{or}}}\,{{{\rm{PCC}}}}(Q,i) \, < \, {{{\rm{PCC}}}}(P,Q)\\ 0 	 {{\mbox{others}}}\hfill\end{array}\right.\\ {{{\rm{CSI}}}}(P,Q)=	 \frac{\mathop{\sum}\limits_{i}{m}_{i}^{P,Q}}{{(N-2)}^{2}}$$

*N* stands for the number of regulons.

Hierarchical clustering with complete linkage was performed based on the CSI matrix to identify different regulon modules. Additionally, 5 was selected as a cutoff for the regulon association network in order to examine the connection between various regulons. The relationship between different regulons was visualized by Cytoscape (version 3.8.0)^95^. The activity score associated with a cell type for each regulon module is defined as the average of the activity scores of its regulon members in all cells within that cell type. The result was then displayed on a UMAP plot.

### Quantifying cluster relationship

The association between various cell types was assessed based on the similarity of total regulon activities, which was defined by the PCC, using the gene regulatory network analysis as a reference (Supplementary Data 22). The results were visualized with Cytoscape (version 3.8.0).

### Identification of mutant

We generated *mybr29* CRISPR-Cas9 transgenic lines using the simplex strategy, as previously described^99^. Specifically, the 20-bp target editing sequence is located within the first exon of MYBR29 (GAGTGTCTCCGAGATCAAGA). Agrobacterium-mediated maize transformation was employed, following the same procedure as for the simplex strategy. To ensure genetic stability, both *mybr29-1* and *mybr29-2* were backcrossed to the B104 genetic background for at least two generations. Lu et al.^100^ identified two single mutants, *mybr19* and *ereb108*, in a large population of sequence-indexed mutations resulting from EMS mutagenesis. These mutants can be obtained through the website http://elabcaas.cn/memd/. Segregating F_2_ ears were utilized in this study. The CRISPR-Cas9-targeted site and EMS-targeted site were amplified from genomic DNA with specific primers (Supplementary Data 26), and the PCR product was analyzed by Sanger sequencing for genotyping.

### MP3RNA-seq and qRT-PCR of mutant

At 8 DAP, 5–10 maize endosperms of the wild type and mutant of the three genes were collected, and total RNA was extracted using the RNAprep Pure Plant Kit (cat. no. DP441, TIANGEN). Three biological replicates were extracted from the kernels of three independent maize ears library construction using the MP3RNA-seq method^101^. cDNA libraries were constructed following Illumina standard protocols and sequenced on the NovaSeq platform (Illumina) by the Berry Genomics company (Beijing) to produce 150-bp paired-end reads. qRT-PCR was performed using SuperReal PreMix Plus (SYBR Green; TIANGEN, Beijing, China; cat. no. FP205) on a CFX96 Real-Time PCR System (Bio-Rad, Hercules, CA, USA) to analyze gene-expression levels. Three independent RNA samples from the kernels of three F_2_ ears were used as biological replicates to ensure reproducibility. The expression of maize 18s was used as an internal control for normalization of gene-expression data. The 2^−ΔΔCt^ method was used to calculate the relative gene-expression level^102^. The primer sequences used for qRT-PCR are provided in Supplementary Data 26.

### MP3RNA-seq analysis

The analysis method is as described in the previous^101^. In brief, raw sequencing reads were sorted into different libraries based on the index read and further separated into different samples based on barcode sequences. Reads were aligned to the respective reference genomes using Hisat2 (version 2.2.1)^103^ software, and duplicates were removed using UMI sequences. CPM was used to measure gene-expression levels, we removed the genes with expression values greater than 50%, differentially expressed genes (*q*-value < 0.05) between the samples were identified using the R package edgeR^92^ (version 3.34.0).

### Cell wall staining

Cell wall staining was performed as described^24^. Briefly, we prepared sections of the 10 DAP maize seed mutant and wild type, the sections were subjected to cellulose staining using 0.1% calcofluor Fluorescent Brightener 28 (Sigma-Aldrich), a commonly used fluorescent dye for cellulose detection. Upon excitation by 405 nm light, fluorescence signal was detected under a Zeiss LSM880 confocal microscope, cellulose-containing primary walls exhibit dark blue fluorescence, while newly deposited wall thickenings appear as light blue fluorescence. This staining method allowed for visualization and localization of cellulose in the plant cell walls.

### Statistical analysis

Statistical analyses for Figs. 3f, 7b, h, i, j, Supplementary Figs. 16e, and 20d, g, h were conducted using a two-tailed Student’s t-test via GraphPad Prism 9, with no adjustments for multiple comparisons. For Supplementary Fig. 14d, the hypergeometric test was employed; Supplementary Fig. 14e utilized the χ2 test, and Supplementary Fig. 20c applied Fisher’s exact test, all performed using R.

### Reporting summary

Further information on research design is available in the Nature Portfolio Reporting Summary linked to this article.

### Supplementary information

Supplementary Information

Peer Review File

Description of Additional Supplementary Files

Supplementary Data 1

Supplementary Data 2

Supplementary Data 3

Supplementary Data 4

Supplementary Data 5

Supplementary Data 6

Supplementary Data 7

Supplementary Data 8

Supplementary Data 9

Supplementary Data 10

Supplementary Data 11

Supplementary Data 12

Supplementary Data 13

Supplementary Data 14

Supplementary Data 15

Supplementary Data 16

Supplementary Data 17

Supplementary Data 18

Supplementary Data 19

Supplementary Data 20

Supplementary Data 21

Supplementary Data 22

Supplementary Data 23

Supplementary Data 24

Supplementary Data 25

Supplementary Data 26

Supplementary Data 27

Reporting Summary

### Source data

Source Data

## Data Availability

The scRNA-seq, bulk RNA-seq and DAP-seq source data are deposited at the Gene Expression Omnibus website (https://www.ncbi.nlm.nih.gov/geo/) under the SuperSeries accession number GSE201701. A genome browser displaying mapped reads is available at https://dap-seq.maize-endosperm.cn/. To facilitate researchers, we have provided a list correlating gene symbols with their respective gene IDs (Supplementary Data 27). Source data are provided with this paper.

## Code availability

The codes used in this article can be accessed at https://github.com/Huo-qiang/maize-endosperm-scRNA-seq^104^.
