# Supplementary material for: Decoding the gene regulatory network of endosperm differentiation in maize
Source: Nat Commun. 2024 Jan 2;15:34. doi: 10.1038/s41467-023-44369-7 (PMC10762121; doi:10.1038/s41467-023-44369-7)
Supplement: Supplementary file 1 — Supplementary Information [file 41467_2023_44369_MOESM1_ESM.pdf]

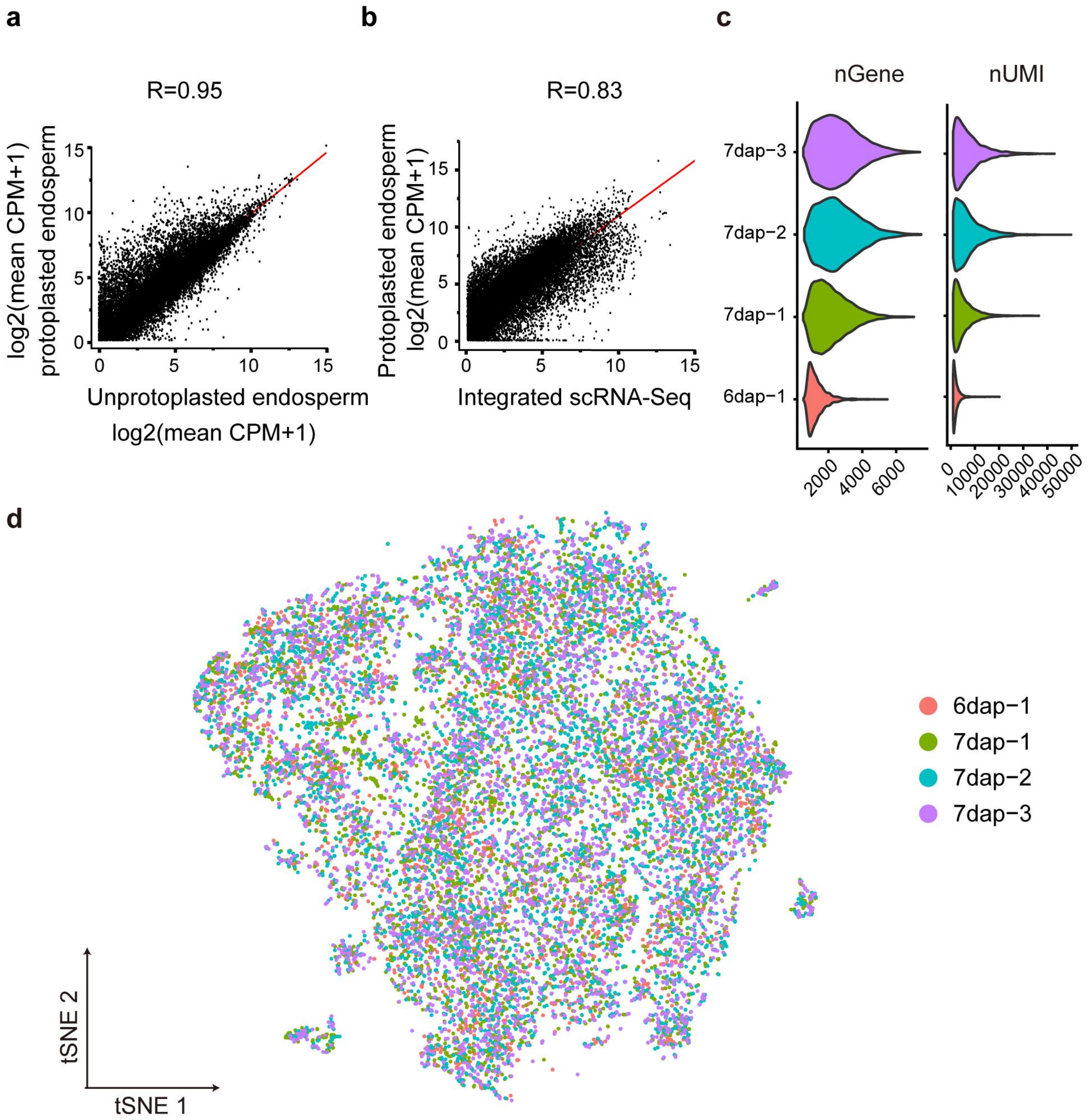

Supplementary Fig. 1. Summary of the maize endosperm scRNA-seq. a, Correlation between the bulk RNA-seq gene expression measurements before and after protoplasting. **b,** Correlation between the merged single-cell and bulk RNA-seq measurements of gene expression from the protoplasts. **c, d,** Quality control parameters (gene and read counts) (c) and tSNE plot (d) for all samples sequenced.

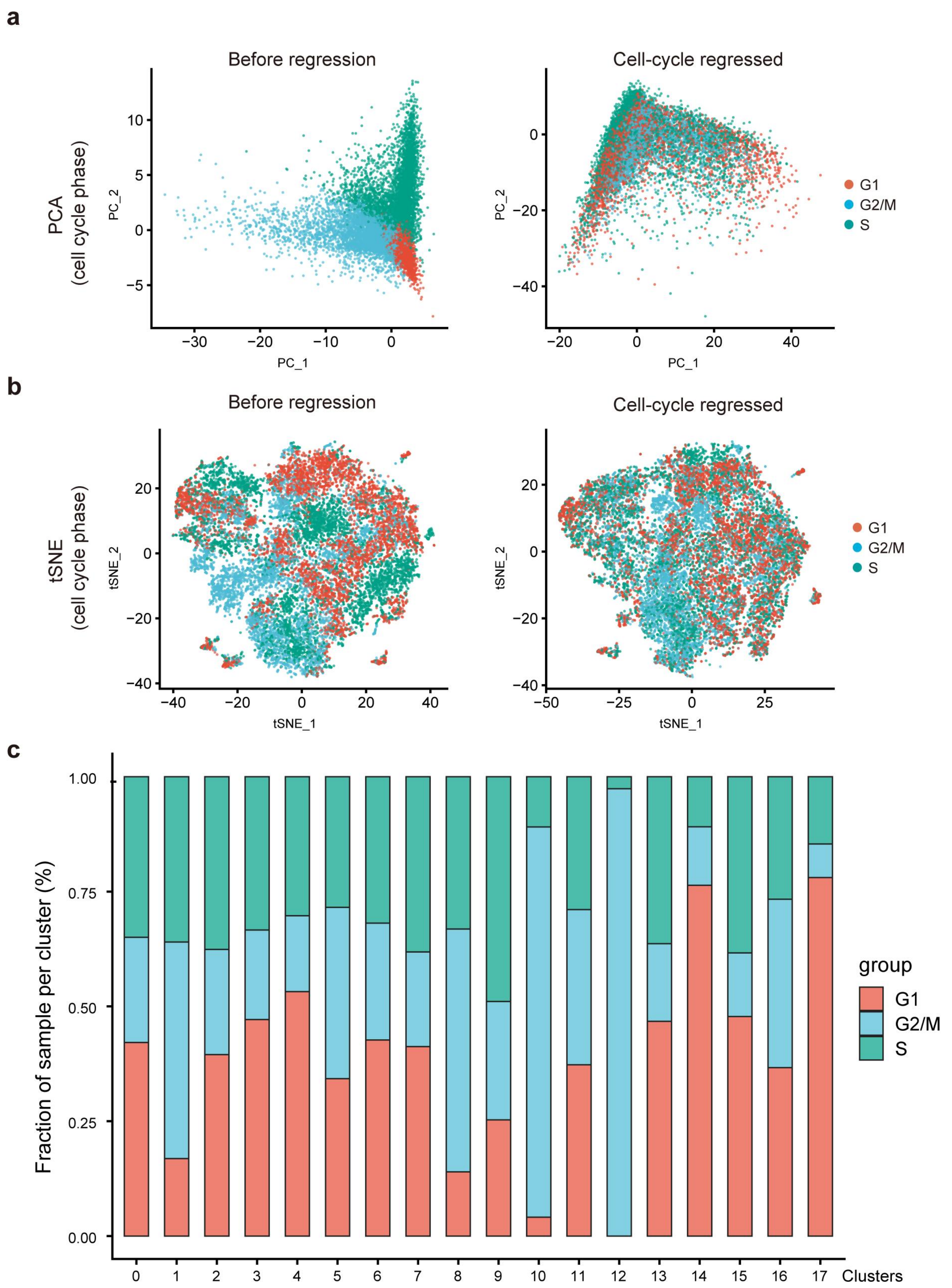

Supplementary Fig. 2. Cell clustering is not driven by the cell cycle. a, PCA plots show the predicted cell cycle phases before and after regression cell cycle. **b,** tSNE plots show the predicted cell cycle phases before and after regression cell cycle. **c,** The bar plot shows the proportion of cells in each stage per cluster after regression cell cycle.

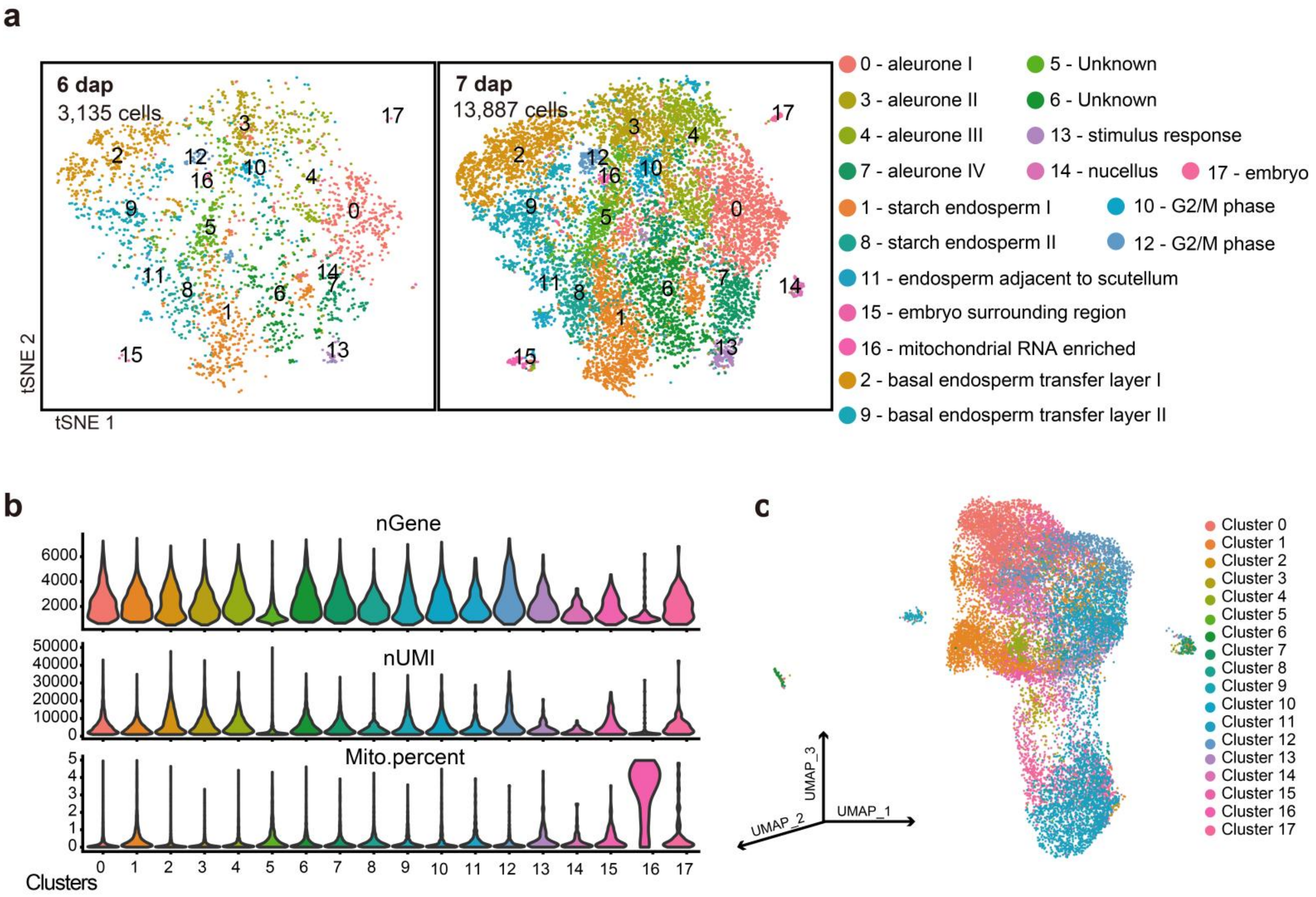

Supplementary Fig. 3. Summary of the cell clusters. a, Clustering analysis identifying 18 clusters from the developing maize endosperm at 6 and 7 DAP. **b,** Quality control parameters (gene and read counts) for the identified 18 clusters. **c,** Visualization of cell clusters by 3D UMAP scatterplots.

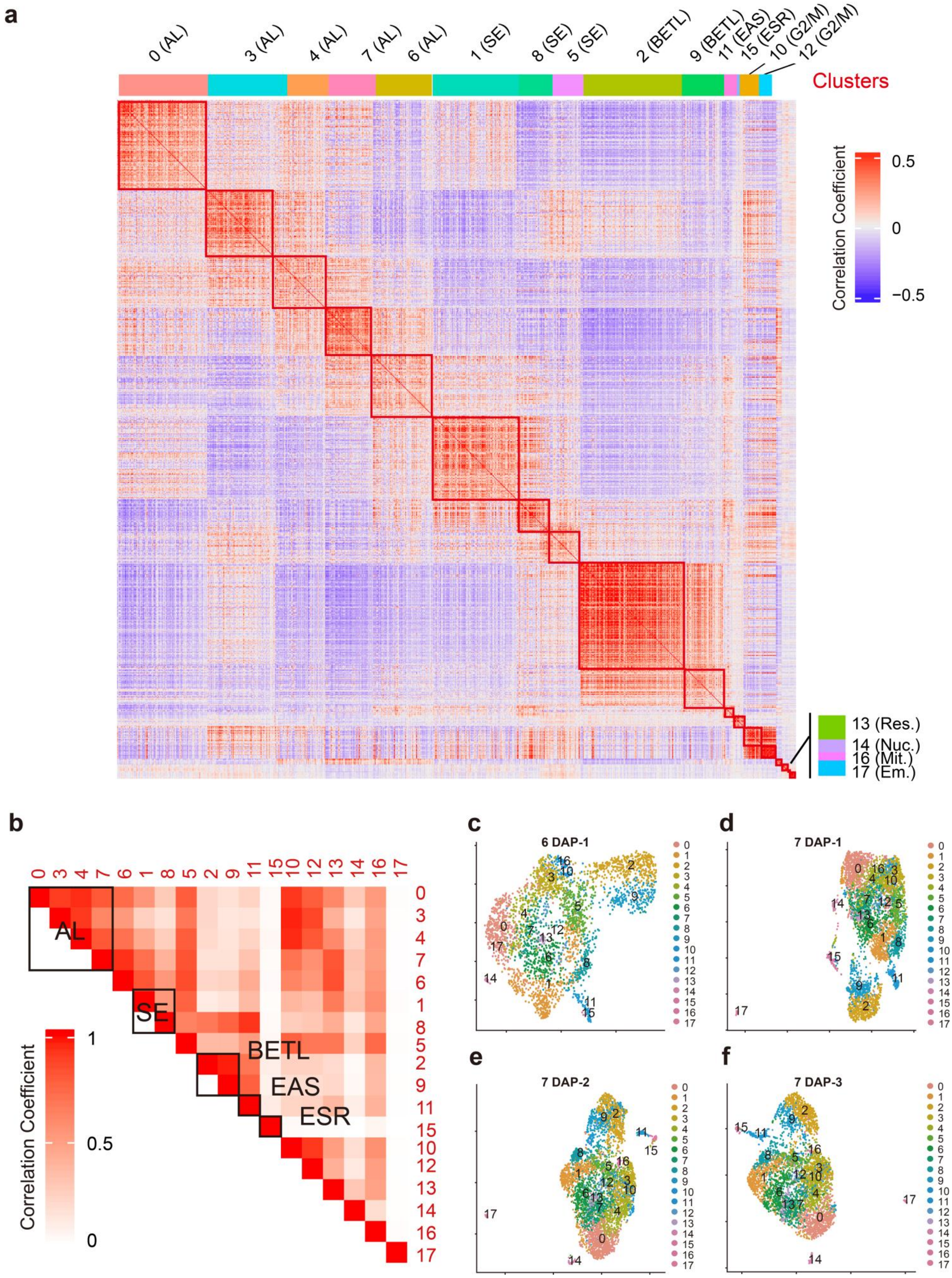

Supplementary Fig. 4. Cell clustering in each individual replicate sample. a, Heatmap showing the correlation between metacells calculated with 2000 highly variable genes. **b,** Heatmap showing the correlation between cell clusters calculated with 2000 highly variable genes. **c-f,** UMAP plots shows the clustering of 4 individual data sets.

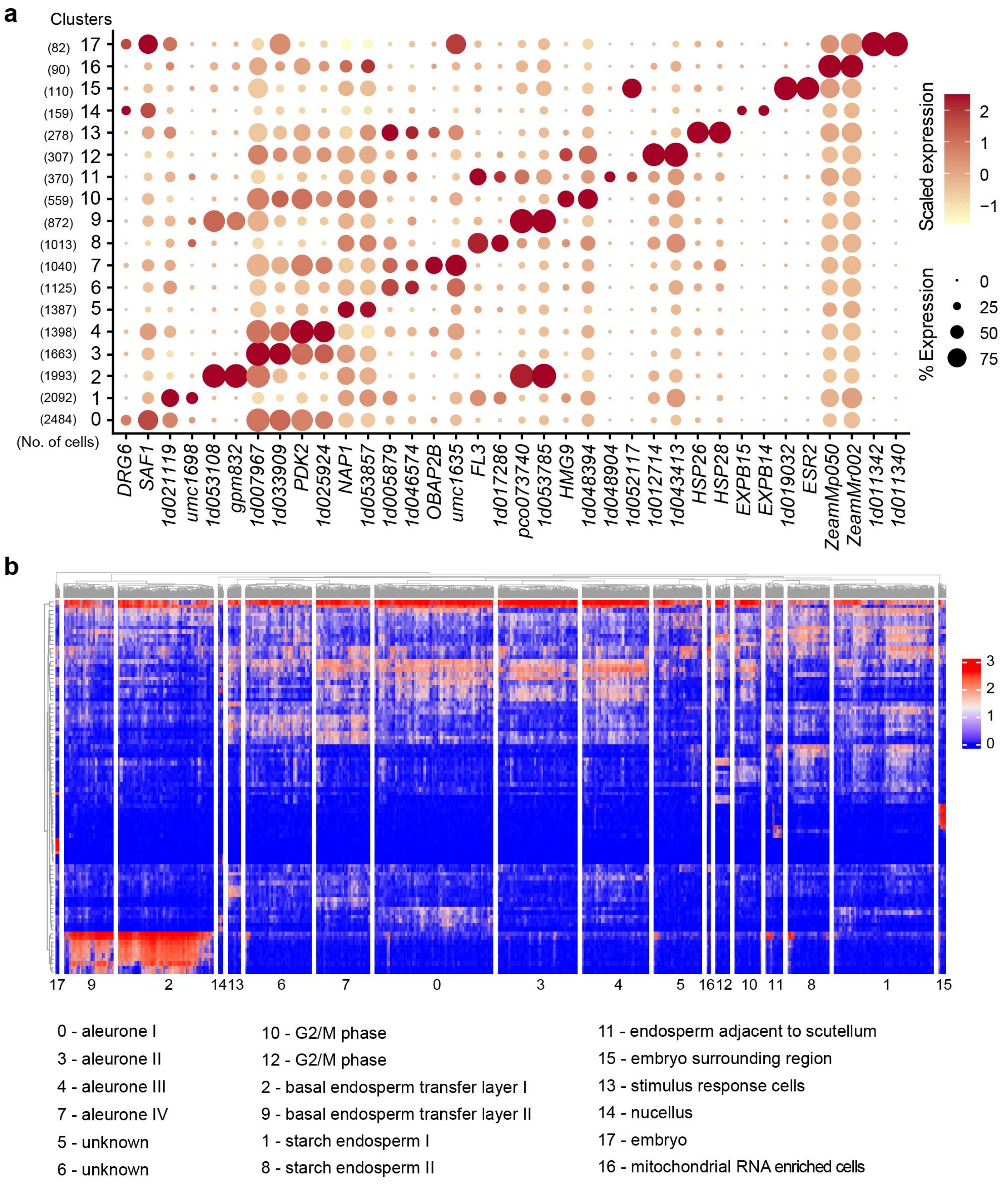

Supplementary Fig. 5. Identification of the cell cluster enriched genes. a, Dot plot of the top two enriched genes identified by differential gene expression analysis for each cluster. Circle size indicates the percentage of cells expressing the marker and color represents the scaled expression value. **b,** Heatmap depicting expression patterns of identified marker genes for 18 clusters. The colors from blue to red represent low to high expression levels.

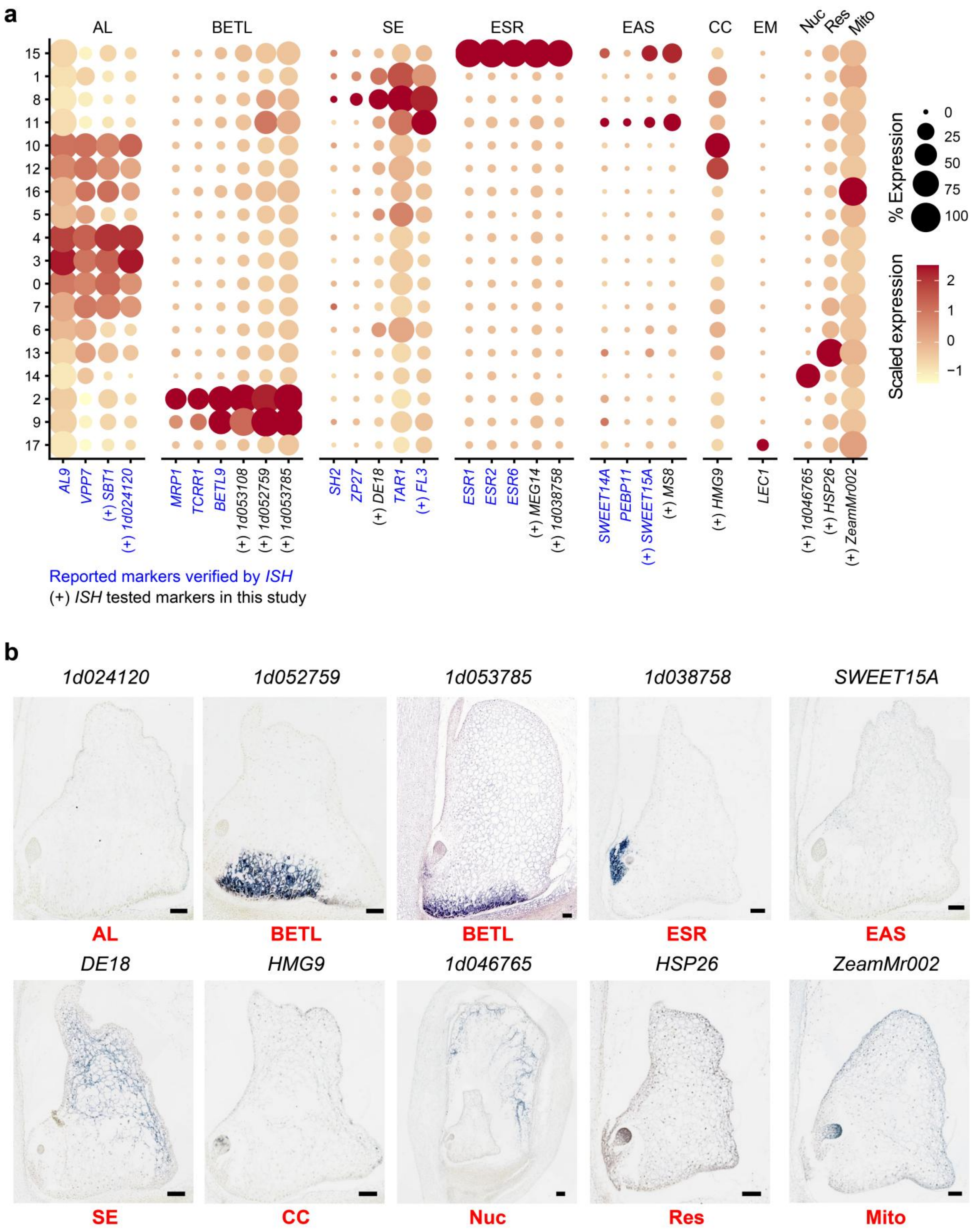

Supplementary Fig. 6. Prediction and validation of the cell identity of each cluster. a, Dot plot of the selected 29 reported marker genes in different maize endosperm cell types. Aleurone (AL), basal endosperm transfer layer (BETL), embryo surrounding region (ESR), endosperm adjacent to scutellum (EAS), starch endosperm (SE), embryo (EM), CC (Cell Cycle), nucellus (Nuc), stimulus response (Res), mitochondrial RNA enriched (Mito). **b,** mRNA *in situ* hybridization (ISH) of selected marker genes validates the predicted cell types of each cluster; scale bars, 100 μm. Experiments were repeated three times with similar results.

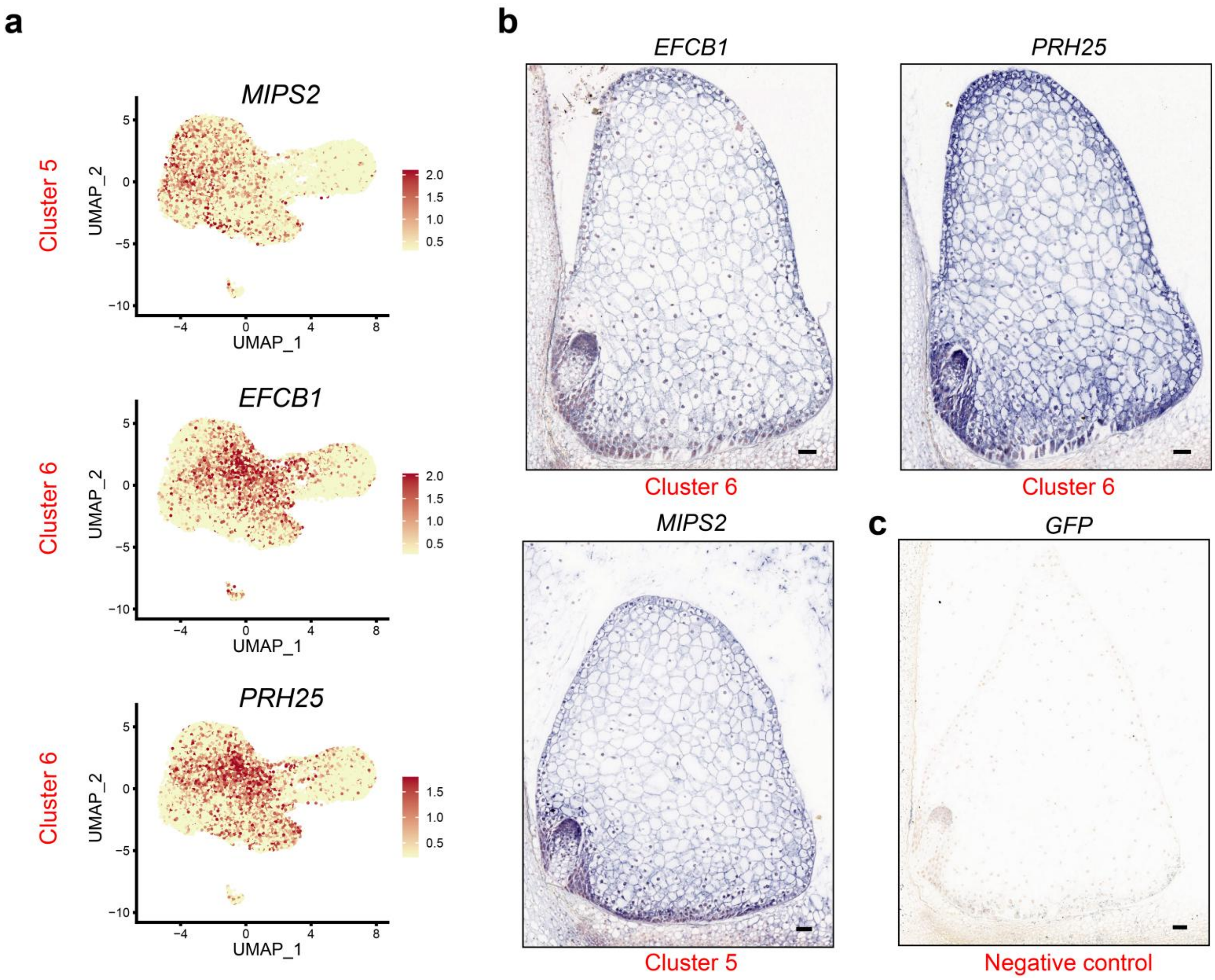

Supplementary Fig. 7. Validation of the cell identity of cluster 5 and 6. a, Feature plots of the selected marker genes in cluster 5 and 6. **b,** mRNA in situ hybridization (ISH) of cluster 5 and 6 marker genes; scale bars, 100 μm. Experiments were repeated three times with similar results. **c,** mRNA in situ hybridization (ISH) of *GFP*; scale bars, 100 μm. Experiments were repeated three times with similar results.

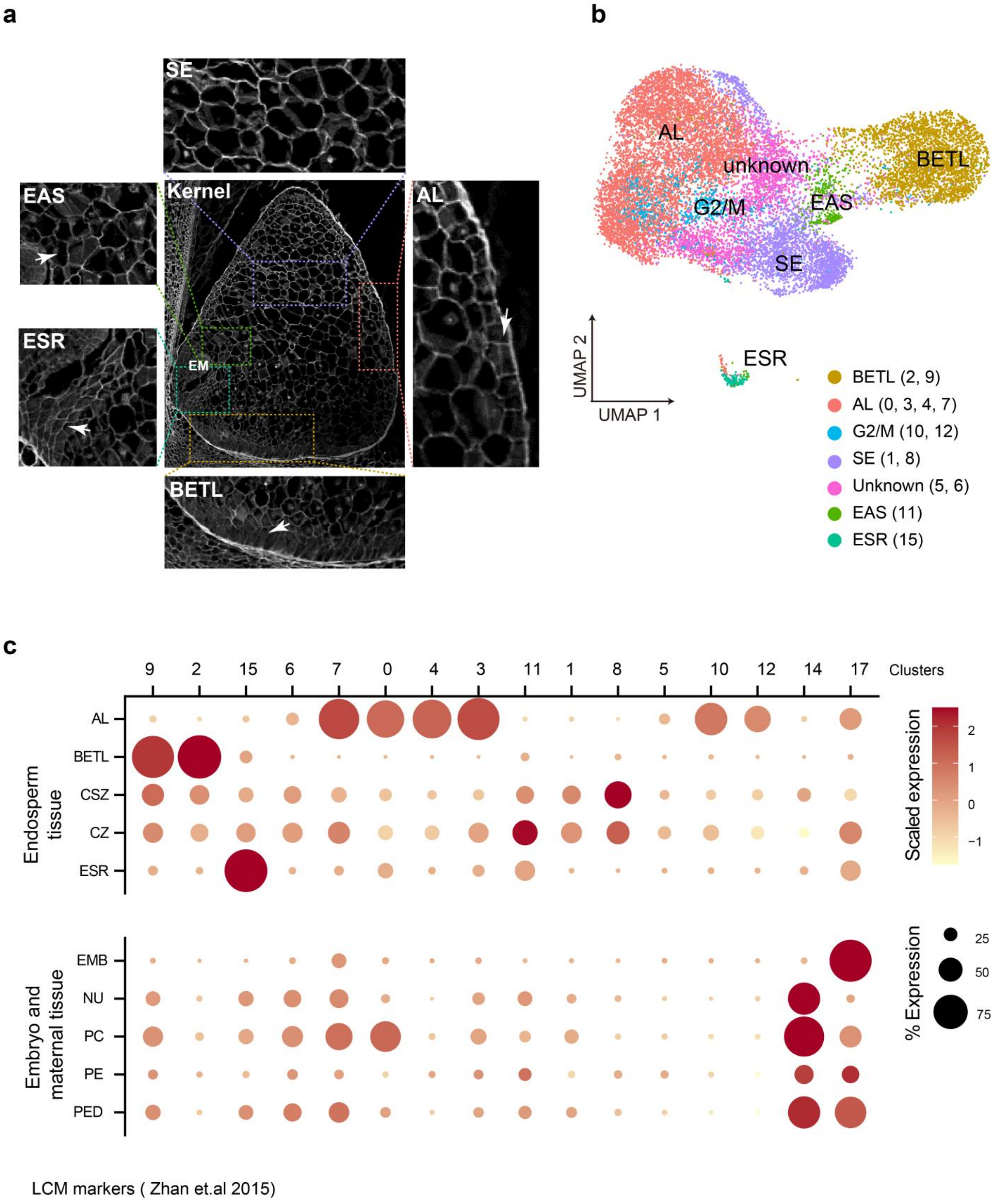

Supplementary Fig. 8. Summary of cell clusters assigned to maize endosperm cell types. a, b, Confocal micrographs (a) of a longitudinal section of maize endosperm during the differentiation stage (on 7 DAP) and UMAP plot (b) of cells shows them grouped into maize endosperm cell types and cell cycle status. Aleurone (AL), basal endosperm transfer layer (BETL), embryo surrounding region (ESR), endosperm adjacent to scutellum (EAS), starch endosperm (SE), embryo (EM), G2/M phase (G2/M). **c,** Dot plot showing the expression of the cell type-specific markers identified from published LCM of sorted cell types in the clusters of our reference atlas. Aleurone (AL), basal endosperm transfer layer (BETL), central starchy endosperm (CSE), conducting zone (CZ), embryo surrounding region (ESR), embryo (EMB), nucellus (NU), placento-chalazal region (PC), pericarp (PE), vascular region of the pedicel (PED).

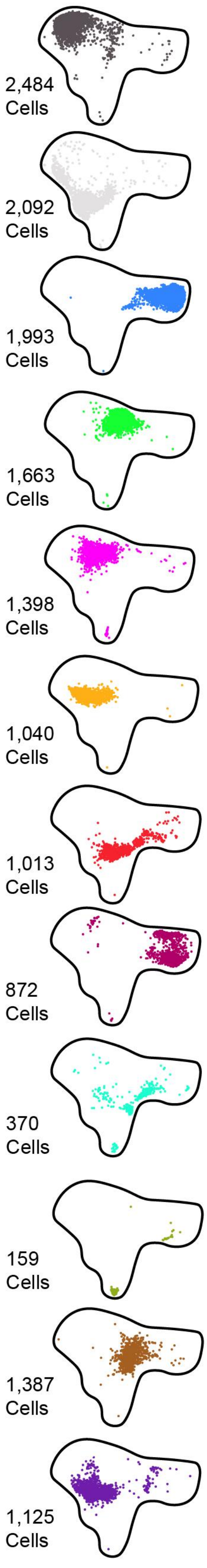

Cluster	GO terms	GO categary	P value	Marker genes		
Cluster 0	nucleosome	GO:CC	2.54E-18	428 genes		19 TFs
	DNA packaging complex	GO:CC	9.63E-18	HON110	TRH3	MYBR40
	protein heterodimerization activity	GO:MF	2.11E-16	FDX3	AAAP60	NACTF30
	nucleoside diphosphate phosphorylation	GO:BP	5.32E-12	GSR2	HON101	WRKY93
	nucleotide phosphorylation	GO:BP	8.05E-12	H3C2	IDP774	MAB16
	oxidation-reduction process	GO:BP	1.58E-11	METS1	TRPS15	WRKY48
	ADP metabolic process	GO:BP	1.59E-11			
	ATP generation from ADP	GO:BP	1.59E-11			
	glycolytic process	GO:BP	1.59E-11			
	chromatin	GO:CC	1.84E-11			
Cluster 1	amide biosynthetic process	GO:BP	1.78E-21	581 genes		35 TFs
	peptide biosynthetic process	GO:BP	2.17E-21	PME26	FIE1	MYO2
	translation	GO:BP	3.90E-21	MPK5	IDP772	NACTF130
	peptide metabolic process	GO:BP	1.28E-20	RHN1	pco069828	THX47
	cellular amide metabolic process	GO:BP	3.44E-20	SRO2	umc1481	WRKY48
	structural molecule activity	GO:MF	1.23E-19	ALT4	1d044698	FL3
	ribonucleoprotein complex	GO:CC	1.56E-17			
	cytosol	GO:CC	1.69E-16			
	cytosolic ribosome	GO:CC	9.73E-16			
	structural constituent of ribosome	GO:MF	1.01E-15			
Cluster 2	structural constituent of ribosome	GO:MF	3.27E-36	386 genes		17 TFs
	peptide metabolic process	GO:BP	8.52E-35	SODH1	BETL3	DOF36
	structural molecule activity	GO:MF	9.49E-34	RPS8	BAP2	MYBR29
	cellular amide metabolic process	GO:BP	1.54E-32	GOT3	BETL10	TCRR2
	translation	GO:BP	2.49E-31	AY105746	BETL9	TCRR1
	cytosolic ribosome	GO:CC	5.09E-31	ELFA12	MEG13	MRP1
	peptide biosynthetic process	GO:BP	6.53E-31			
	amide biosynthetic process	GO:BP	5.37E-30			
	ribosome	GO:CC	5.96E-30			
	ribosomal subunit	GO:CC	1.33E-28			
Cluster 3	mitochondrion	GO:CC	2.26E-97	523 genes		3 TFs
	mitochondrial envelope	GO:CC	6.54E-93	NAD2	VPP8	VPP10
	mitochondrial membrane	GO:CC	9.53E-89	SODH1	GPC2	PLATZ2
	mitochondrial protein-containing complex	GO:CC	1.98E-81	MIPS2	PAC1	DBB11
	mitochondrial inner membrane	GO:CC	1.55E-80	gpm642	ALT9	
	organelle inner membrane	GO:CC	9.02E-74	1d036018	ACPT1	
	inner mitochondrial membrane protein complex	GO:CC	9.74E-70			
	respirasome	GO:CC	3.55E-62			
	organelle envelope	GO:CC	1.97E-56			
	envelope	GO:CC	1.97E-56			
Cluster 4	mitochondrial envelope	GO:CC	2.42E-64	411 genes		11 TFs
	mitochondrial membrane	GO:CC	1.35E-62	GL14	LACS2	CRR1
	mitochondrial inner membrane	GO:CC	3.46E-62	AOC1	GRX1	PRH25
	respirasome	GO:CC	8.93E-61	SUS1	CCR2	HSFTF1
	organelle inner membrane	GO:CC	2.95E-57	SBT1	CCR1	ABI36
	inner mitochondrial membrane protein complex	GO:CC	1.59E-53	AL9	NAD6	MYB25
	mitochondrial respirasome	GO:CC	1.08E-51			
	respiratory chain complex	GO:CC	1.53E-50			
	mitochondrial protein-containing complex	GO:CC	1.33E-46			
	mitochondrion	GO:CC	1.94E-44			
Cluster 7	nucleosome	GO:CC	1.06E-40	574 genes		83 TFs
	DNA packaging complex	GO:CC	1.40E-39	CRR1	PDH4	MAB16
	protein heterodimerization activity	GO:MF	6.18E-30	pco061453	1d035542	CRR1
	chromatin	GO:CC	1.32E-27	FAD7	SUDH2	ZIM27
	protein-DNA complex	GO:CC	1.13E-24	MSL5	HON110	NACTF13
	nucleosome assembly	GO:BP	2.92E-19	1d007035	HIS404	GATA18
	chromatin assembly	GO:BP	2.32E-17			
	nucleosome organization	GO:BP	4.78E-17			
	chromatin assembly or disassembly	GO:BP	1.20E-16			
	DNA packaging	GO:BP	5.78E-15			
Cluster 8	structural constituent of ribosome	GO:BP	8.46E-136	437 genes		7 TFs
	structural molecule activity	GO:BP	2.38E-132	CNGT1	AE1	HDT103
	translation	GO:MF	2.53E-130	TAR1	TUA6	HDT102
	ribosome	GO:MF	3.71E-130	HFI1	RFZ1	NACTF130
	peptide biosynthetic process	GO:CC	4.28E-129	FSGT1	TUA5	O11
	amide biosynthetic process	GO:BP	2.48E-126	DE18	O11	FL3
	cytosolic ribosome	GO:BP	4.80E-124			
	peptide metabolic process	GO:CC	4.03E-122			
	ribosomal subunit	GO:CC	5.27E-120			
	cellular amide metabolic process	GO:BP	2.20E-116			
Cluster 9	nucleosome	GO:CC	5.97E-59	238 genes		12 TFs
	DNA packaging complex	GO:CC	9.11E-58	1d006213	MEG13	NACTF23
	protein heterodimerization activity	GO:MF	2.39E-48	HON106A	MEG1	BZIP60
	chromatin	GO:CC	5.26E-45	1d011434	PSEI8	MYB73
	protein-DNA complex	GO:CC	1.16E-40	1d019045	BETL9	MYBR29
	chromosome	GO:CC	2.00E-28	GA2OX6	BAP2	TCRR2
	nucleosome assembly	GO:BP	2.76E-28			
	chromatin assembly	GO:BP	2.51E-26			
	nucleosome organization	GO:BP	5.31E-26			
	chromatin assembly or disassembly	GO:BP	1.38E-25			
Cluster 11	ubiquitin-like protein conjugating enzyme activity	GO:MF	2.66E-09	244 genes		17 TFs
	ubiquitin conjugating enzyme activity	GO:MF	2.18E-08	IPS1	1d010814	NACTF23
	establishment of protein localization to endoplasmic reticulum	GO:BP	3.80E-07	pco097814	XTH5	NACTF49
	protein targeting to ER	GO:BP	3.80E-07	1d025862	CP33C	pco124760
	protein localization to endoplasmic reticulum	GO:BP	9.73E-07	GG2	TUA5	ZIM16
	intracellular transport	GO:BP	1.05E-06	GRP1	CAMTA3	EREB172
	cellular macromolecule localization	GO:BP	2.70E-06			
	intracellular protein transport	GO:BP	3.33E-06			
	establishment of localization in cell	GO:BP	3.77E-06			
	protein polyubiquitination	GO:BP	4.06E-06			
Cluster 15	signal peptide processing	GO:BP	3.72E-10	105 genes		4 TFs
	signal peptidase complex	GO:CC	5.86E-09	ESR2	PSEI8	ARFTF36
	protein processing	GO:BP	1.33E-08	DEF3	MS8	CPP2
	endoplasmic reticulum	GO:CC	1.62E-08	ESR1	MN6	NACTF130
	protein maturation	GO:BP	4.99E-07	ESR6	CAH2	EREB100
	catalytic activity, acting on a protein	GO:MF	3.65E-05	ESR3	JMJ12	
	peptidase activity	GO:MF	9.56E-05			
	endoplasmic reticulum membrane	GO:CC	0.000244019			
	nuclear outer membrane-endoplasmic reticulum membrane network	GO:CC	0.000248688			
	endoplasmic reticulum subcompartment	GO:CC	0.000263173			
Cluster 5	translation	GO:BP	2.41E-75	169 genes		3 TFs
	peptide biosynthetic process	GO:BP	1.29E-74	EIF6	1d004927	HDT102
	amide biosynthetic process	GO:BP	2.11E-72	ARPP40	NTF2	HDT103
	peptide metabolic process	GO:BP	8.73E-70	TPI4	1d007824	DBB11
	cellular amide metabolic process	GO:BP	6.62E-67	TUFM1	MCH1	
	ribosome	GO:CC	5.84E-64	GRP2	RPS13	
	structural constituent of ribosome	GO:MF	1.78E-63			
	structural molecule activity	GO:MF	3.89E-63			
	organonitrogen compound biosynthetic process	GO:BP	2.61E-61			
	ribosomal subunit	GO:CC	1.11E-60			
Cluster 6	cytosolic ribosome	GO:CC	2.32E-11	434 genes		56 TFs
	ribosomal subunit	GO:CC	2.00E-10	OMT2	GTE102	WRKY79
	Ribosome	KEGG	5.51E-10	si660041a03	1d053367	EREB147
	ribosome	GO:CC	5.37E-09	ARPP2A-2	GST48	EREB54
	structural constituent of ribosome	GO:MF	1.48E-08	1d045069	PSEI5	PRH25
	structural molecule activity	GO:MF	3.12E-06	magi93696	1d033402	EREB209
	cytosolic large ribosomal subunit	GO:CC	1.49E-05			
	cytosolic small ribosomal subunit	GO:CC	4.36E-05			
	calcium ion binding	GO:MF	7.72E-05			
	small ribosomal subunit	GO:CC	8.31E-05			

Supplementary Data Fig. 9. GO enrichment and selected marker genes for each cluster. Top 10 GO terms of each cell cluster. GO category, *P* value and selected cluster-enriched genes (TFs) for each item are given. Cells of each cluster highlighted in the UMAP plot are shown on the left.

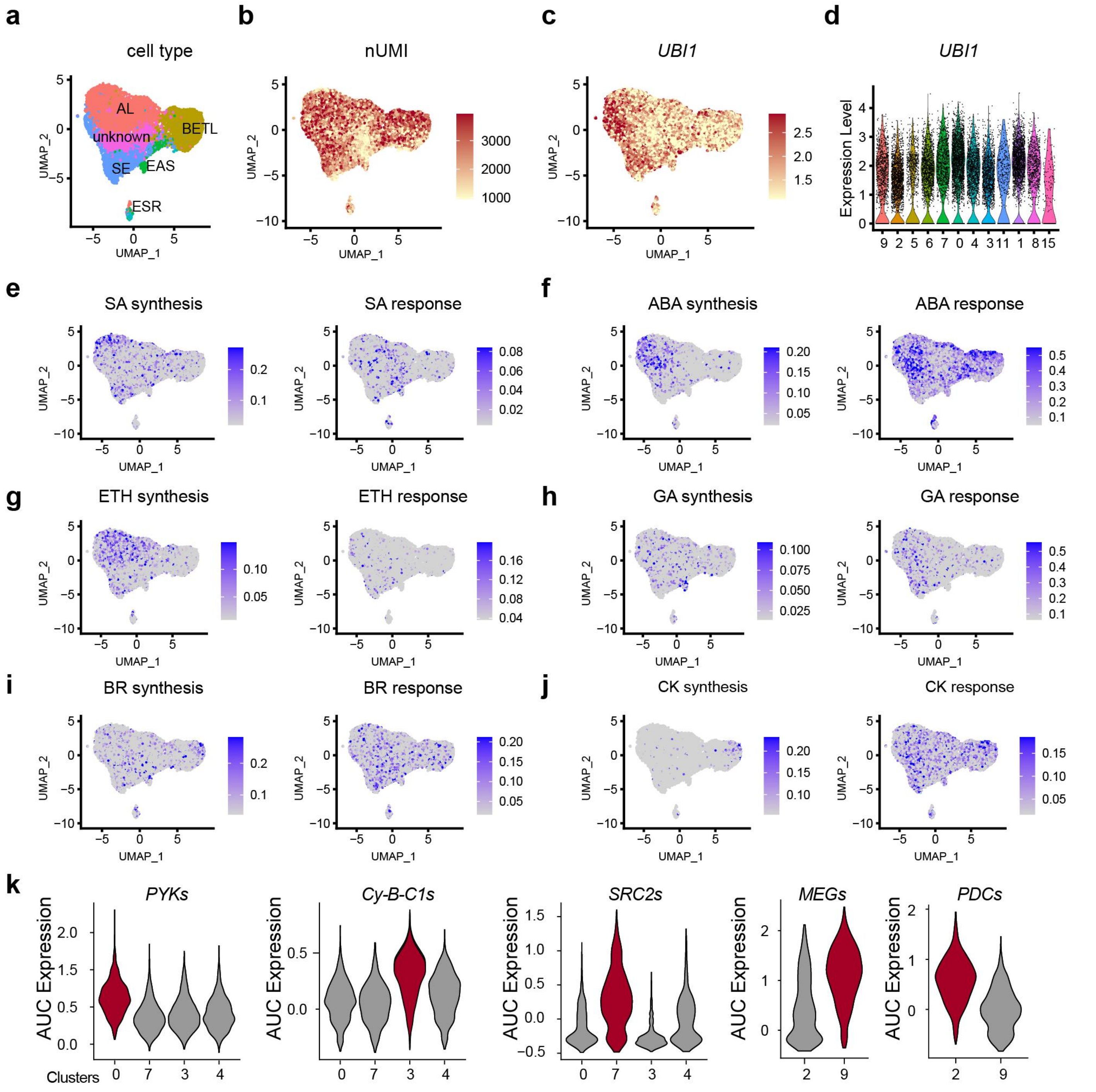

Supplementary Fig. 10. UMAP plots of phytohormone-related genes in each cell cluster. a, UMAP plot of five cell types in maize endosperm. **b,** Feature plot displaying the UMI value for each cell. **c,** Feature plot displaying the expression of an internal control gene, *UBI1*. **d,** Violin plot showing the expression pattern of *UBI1* in 12 cell clusters. **e-j,** UMAP plots of expression patterns of the genes related to salicylic acid (SA), abscisic acid (ABA), ethylene (ETH), gibberellin (GA), brassinosteroid (BR) and cytokinin (CK) biosynthesis and response. The colors from grey to blue represent low to high expression levels from low to high of these genes in individual cells. **k**, Violin plot showing the expression pattern of selected functional gene sets in AL or BETL cell clusters.

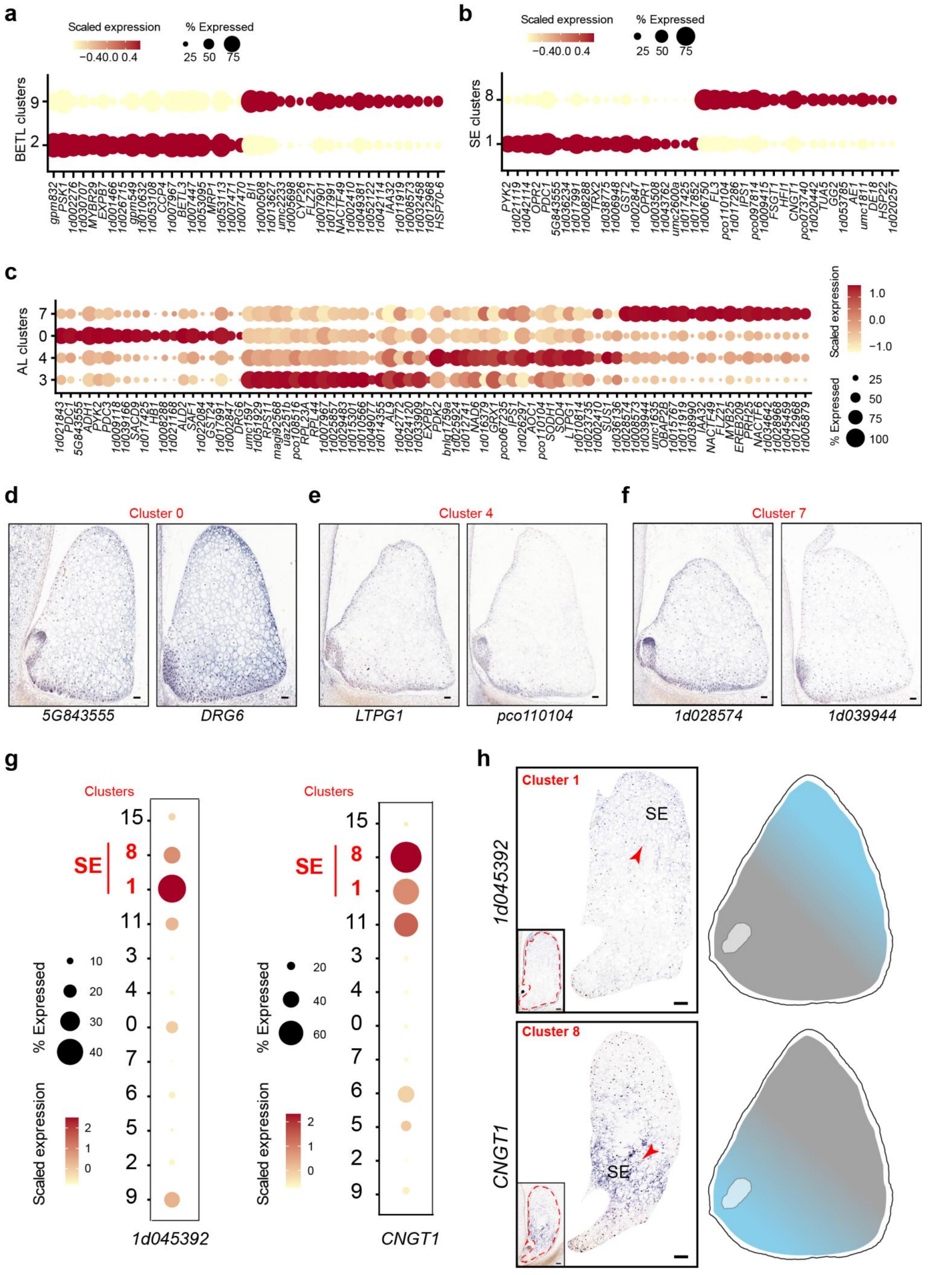

Supplementary Fig. 11. Identification of the cell subclusters-enriched genes. a, Dot plot of the top twenty enriched genes identified by differential gene expression analysis for BETL subclusters. **b,** Dot plot of the top twenty enriched genes identified by differential gene expression analysis for SE subclusters. **c,** Dot

plot of the top twenty enriched genes identified by differential gene expression analysis for AL subclusters. **d-f,** mRNA in situ hybridization (ISH) of selected marker genes of cluster 0, 4, and 7; scale bars, 50 μm. Experiments were repeated three times with similar results. **g,** Dot plots displaying selected markers gene of Cluster 1 and Cluster 8. **h,** mRNA in situ hybridization results of the genes shown in (g); scale bars,100μm. Experiments were repeated three times with similar results.

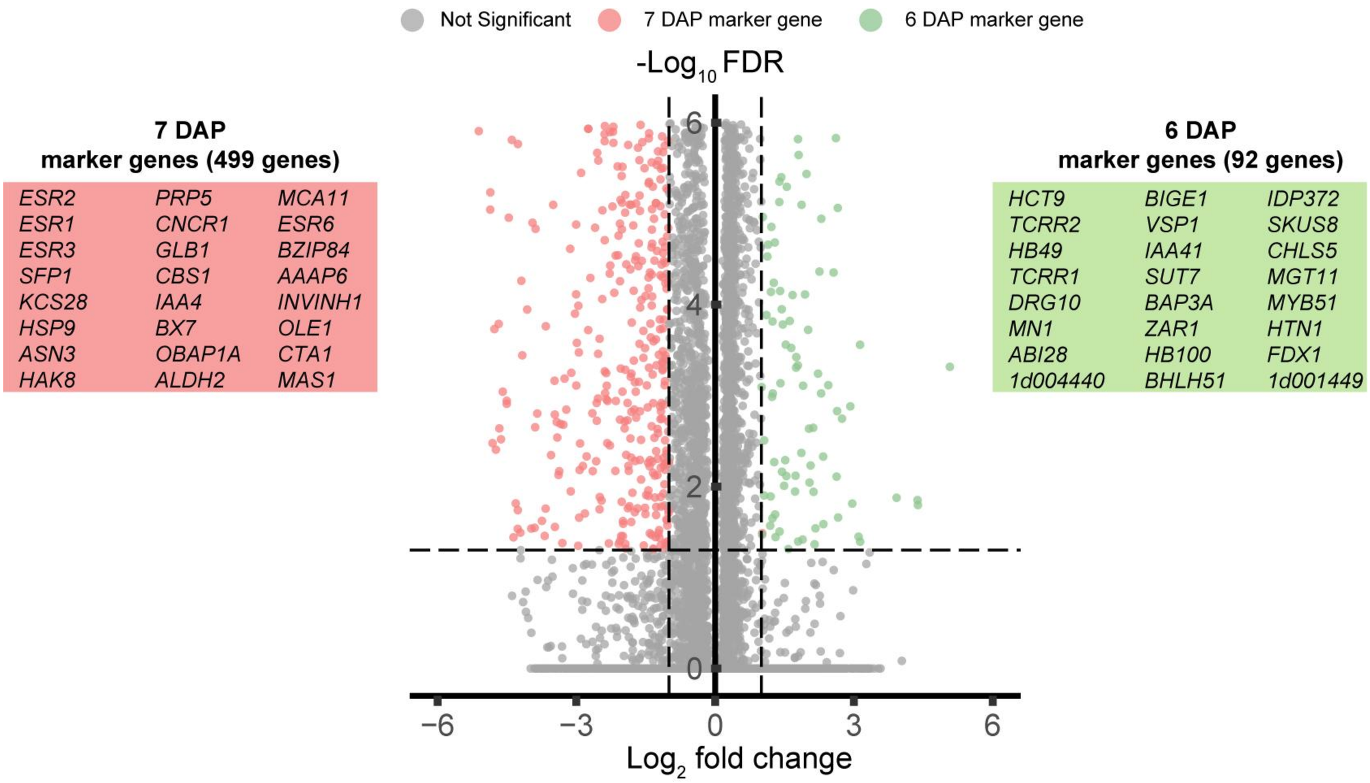

Supplementary Fig. 12. Identification of marker genes between 6 DAP and 7 DAP. Differentially expressed genes between 6 DAP and 7 DAP in bulk RNA-seq. Two dotted vertical lines indicate gene expression fold change (6 DAP vs. 7 DAP) ≥2 and ≤ −2, respectively, and the horizontal dotted line indicates the adjusted *P* value (FDR q-value) of 0.05.

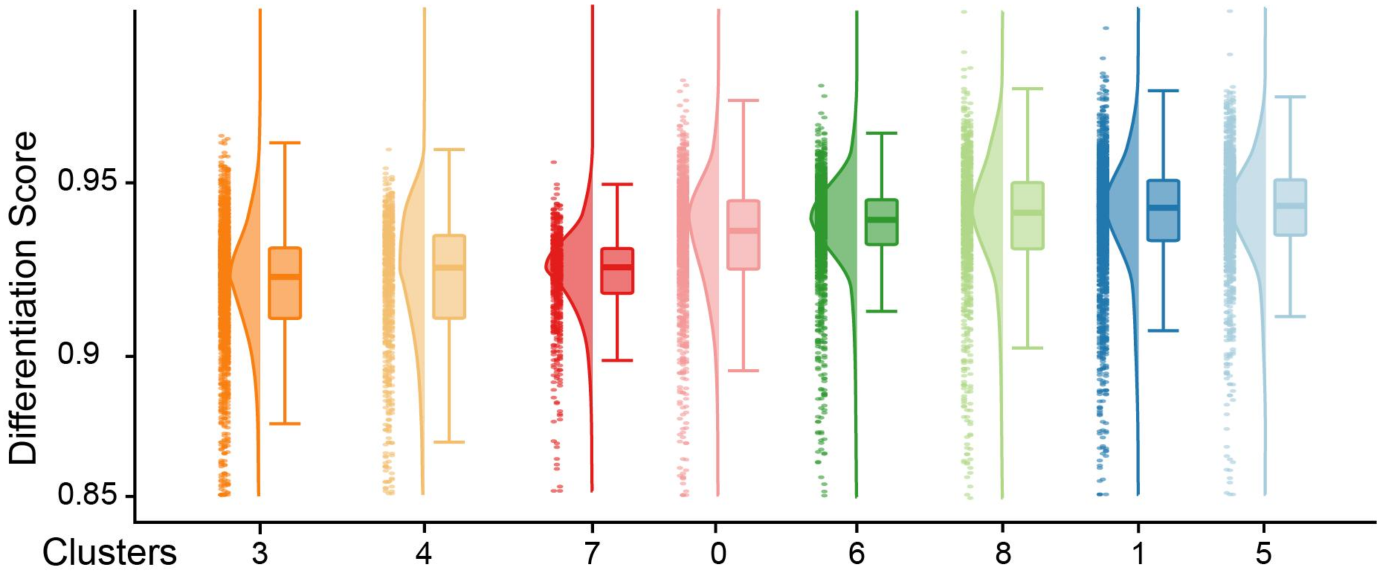

Supplementary Fig. 13. Cell differentiation score. Raincloud plot showing the cell differentiation score of 0, 1, 3, 4, 5, 6, 7 and 8 cluster at 7 DAP. N = 2,043 cells in cluster 0, n = 1,644 cells in cluster 1, n = 1,358 cells in cluster 3, n = 1,216 cells in cluster 4, n = 732 cells in cluster 5, n = 1,161 cells in cluster 6, n = 968 cells in cluster 7. Box plots indicate median (middle line), 25th, 75th percentile (box) and 5th and 95th percentile (whiskers) as well as all data (single points).

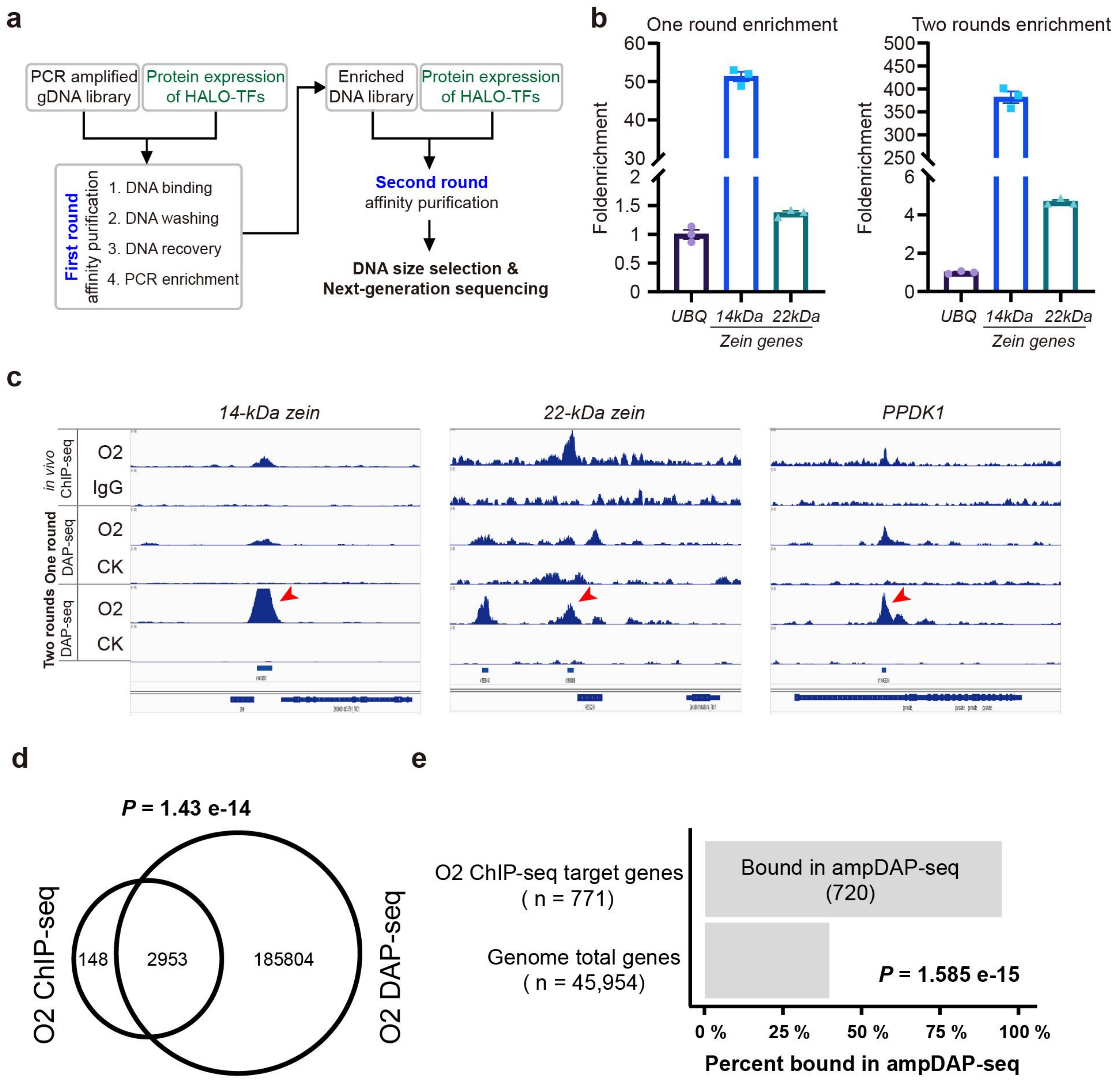

Supplementary Fig. 14. Optimization of ampDAP-seq experiment flow. a, Two-round ampDAP-seq experiment flow. **b,** The histogram shows fold enrichment of different targets of O2. Error bars indicate ±SEM (n = 3 technical replications). Source data are provided as a Source Data file. **c,** IGV genomic track plots illustrating the targeting of target genes by O2. The expected peaks were indicated by red arrows. **d,** Venn Diagram showing the overlap of peaks for ampDAP-seq and ChIP-seq for O2. The hyper geometric test was performed to calculate the *P* value. **e,** O2 ampDAP-seq target genes appeared significantly more frequently in O2 ChIP-seq target gene promoters than in genome-wide gene promoters. The $\chi 2$ test was performed to calculate the *P* value.

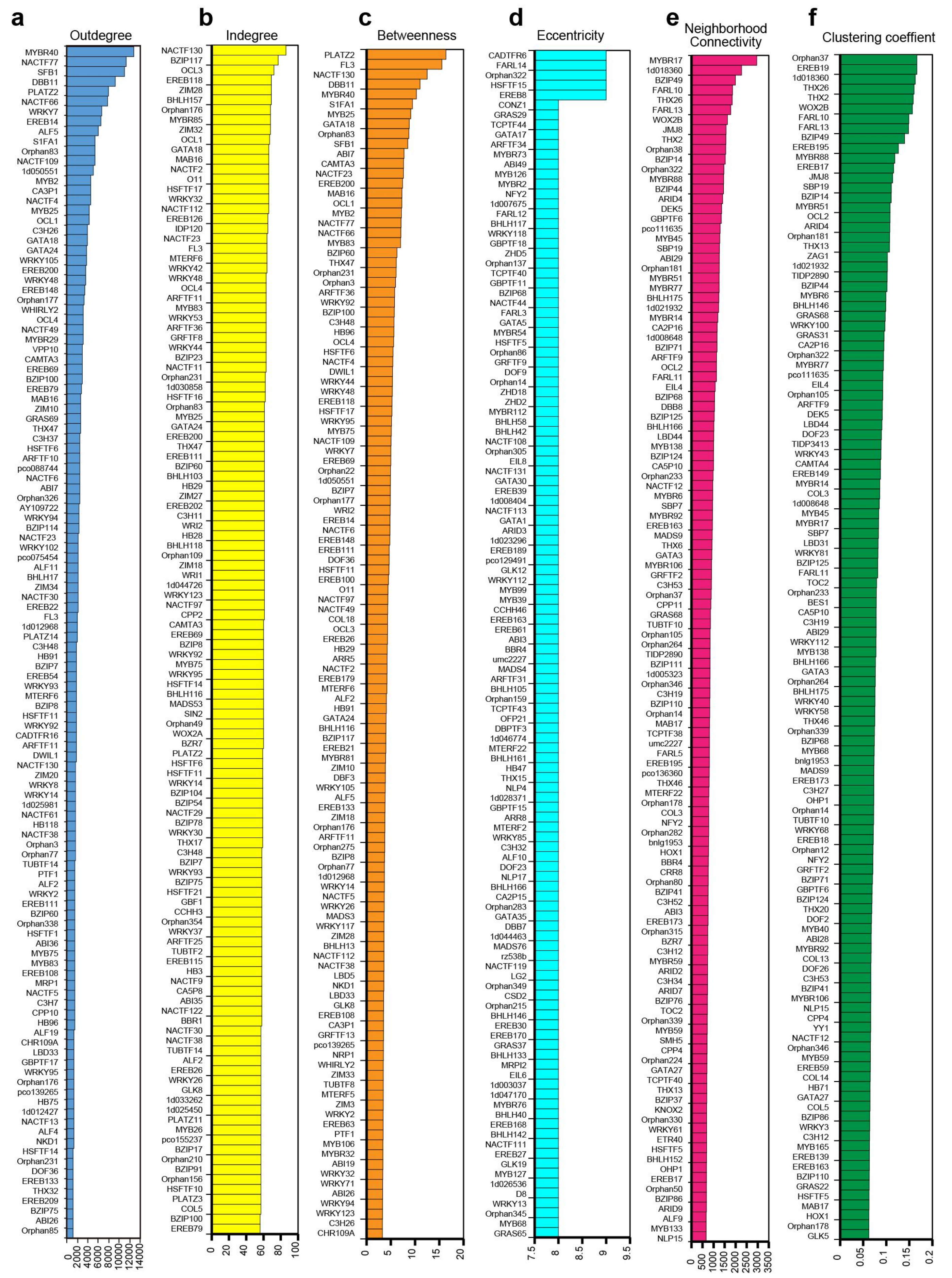

Supplementary Fig. 15. Predicted key regulators based on network topology analysis. The top-ranked TFs in the GRN based on network topology analysis. Out-degree **(a)**; In-degree **(b)**; Betweenness **(c)**; Eccentricity **(d)**; Neighborhood connectivity **(e)**; Clustering coefficient **(f)**.

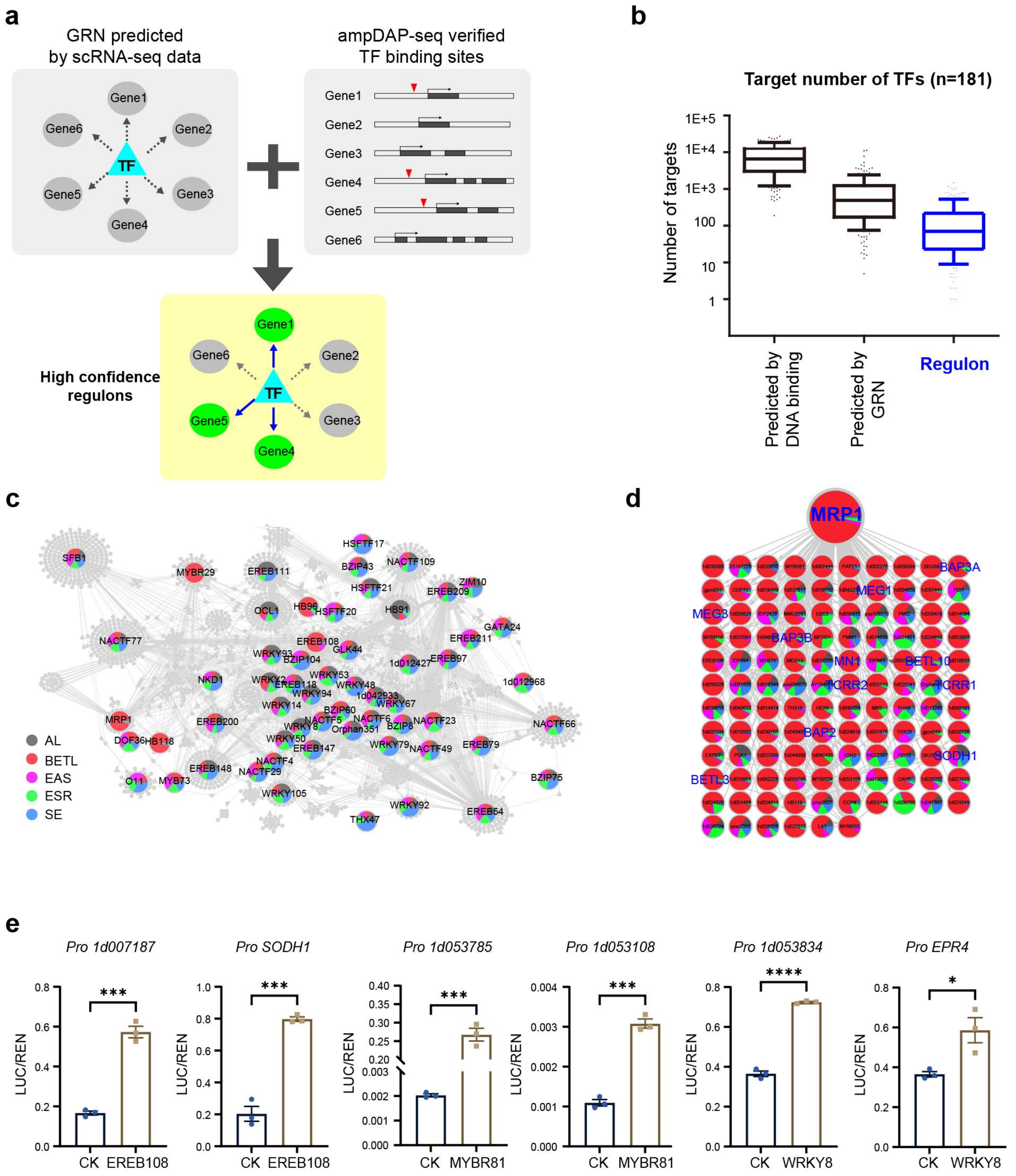

Supplementary Fig. 16. Identifying a regulon using a coexpression GRN combined with TF-binding profiles and validation of our regulon-based GRN. a, A schematic diagram of high-confidence regulon identification. **b,** Box plot of the number of targets of the selected TFs predicted based on the GRN, DNA binding profiles and regulons. Box plots indicate median (middle line), 25th, 75th percentile (box) and 5th and 95th percentile (whiskers) as well as outliers (single points). **c,** Network plot of selected TFs and targets. Colors represent the expression level of TFs in different cell types. **d,** Representative targets of MRP1. Colors represent the expression level in different cell types as shown in (c). **e,** Transactivation assays

confirming the transcription regulatory activities of selected TFs on their targets. Error bars indicate ±SEM (n = 3 technical replications). *, $P < 0.05$, ***, $P < 0.001$, ****, $P < 0.0001$; Two-tailed student's t-test. No adjustments were made for multiple comparisons test. Source data are provided as a Source Data file.

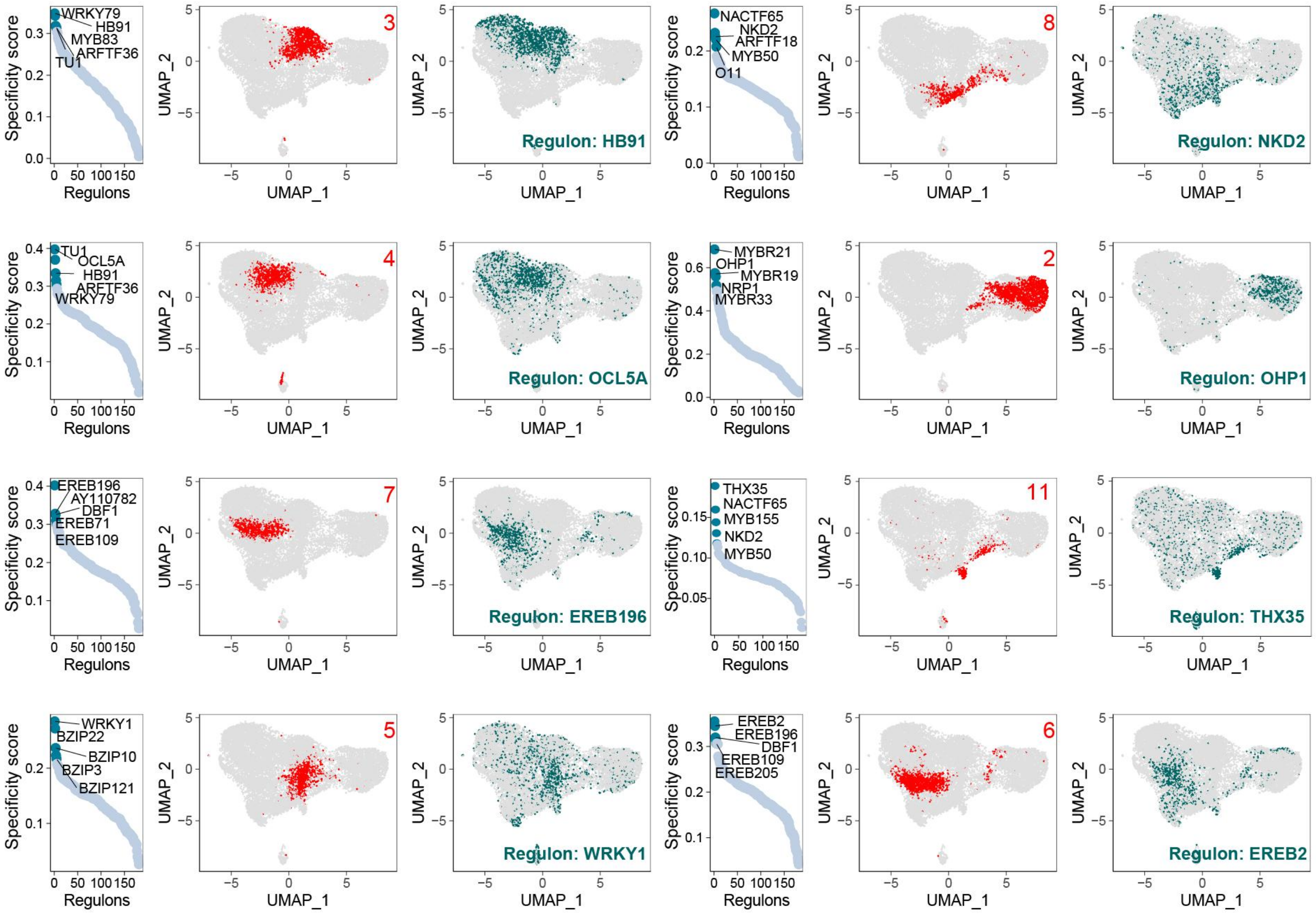

Supplementary Fig. 17. Top5 representative regulon for each cell cluster. Ranking of regulons in each cell cluster based on the regulon specificity score and cell types are highlighted in the UMAP plots (red dots) and binarized regulon activity scores for selected regulons are shown on the UMAP plots (dark green dots).

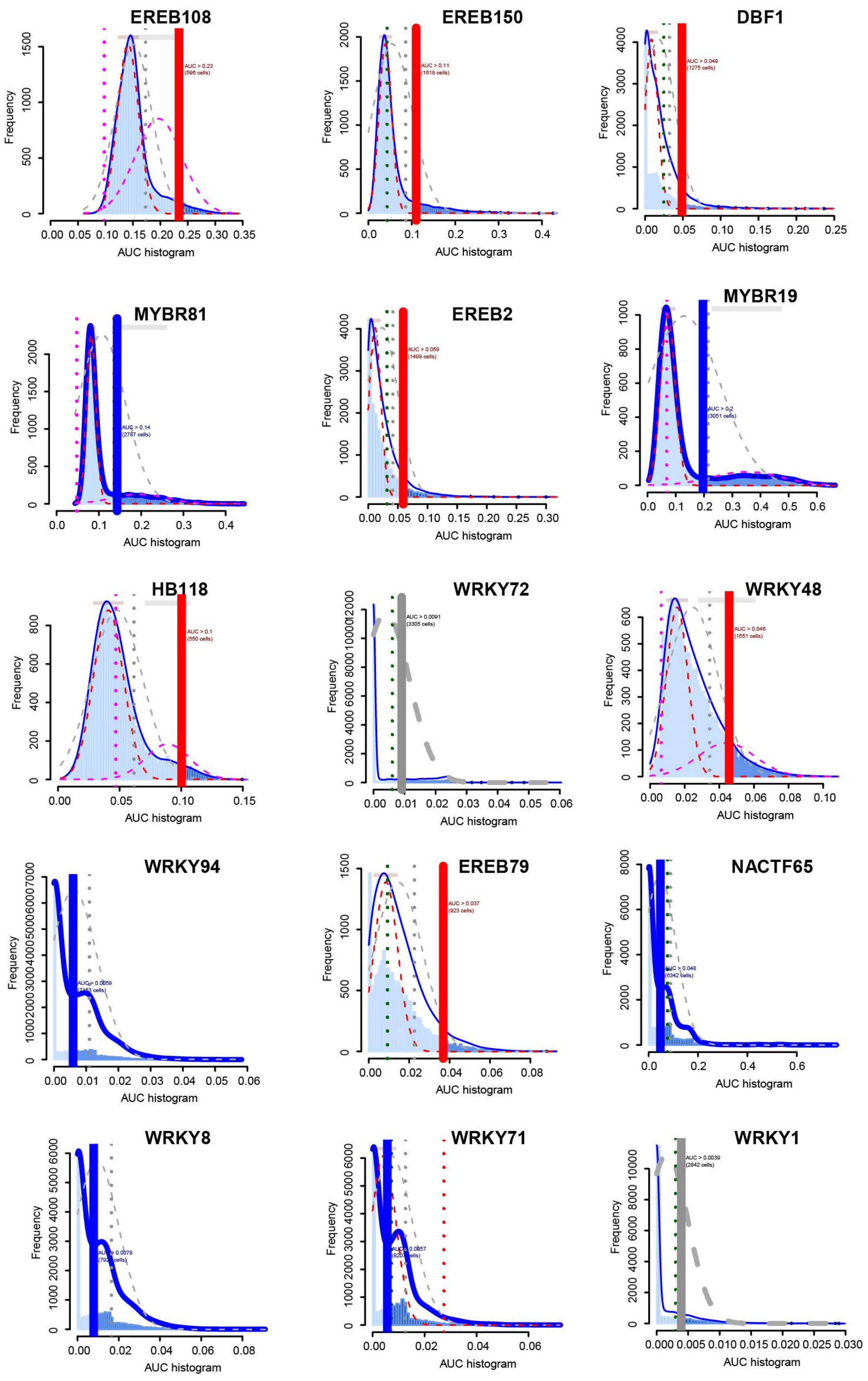

Supplementary Fig. 18. AUCell histograms of selected regulons. AUC distributions for selected regulons. The AUC represents the activity of the regulon or gene signature in each cell. The selection of cells with the regulon "active" is based on the distribution of the AUC across all the cells in the dataset. The ideal situation of a regulon or gene signature being active in only a subset of the cells would return a bimodal distribution or a distribution with a long tail.

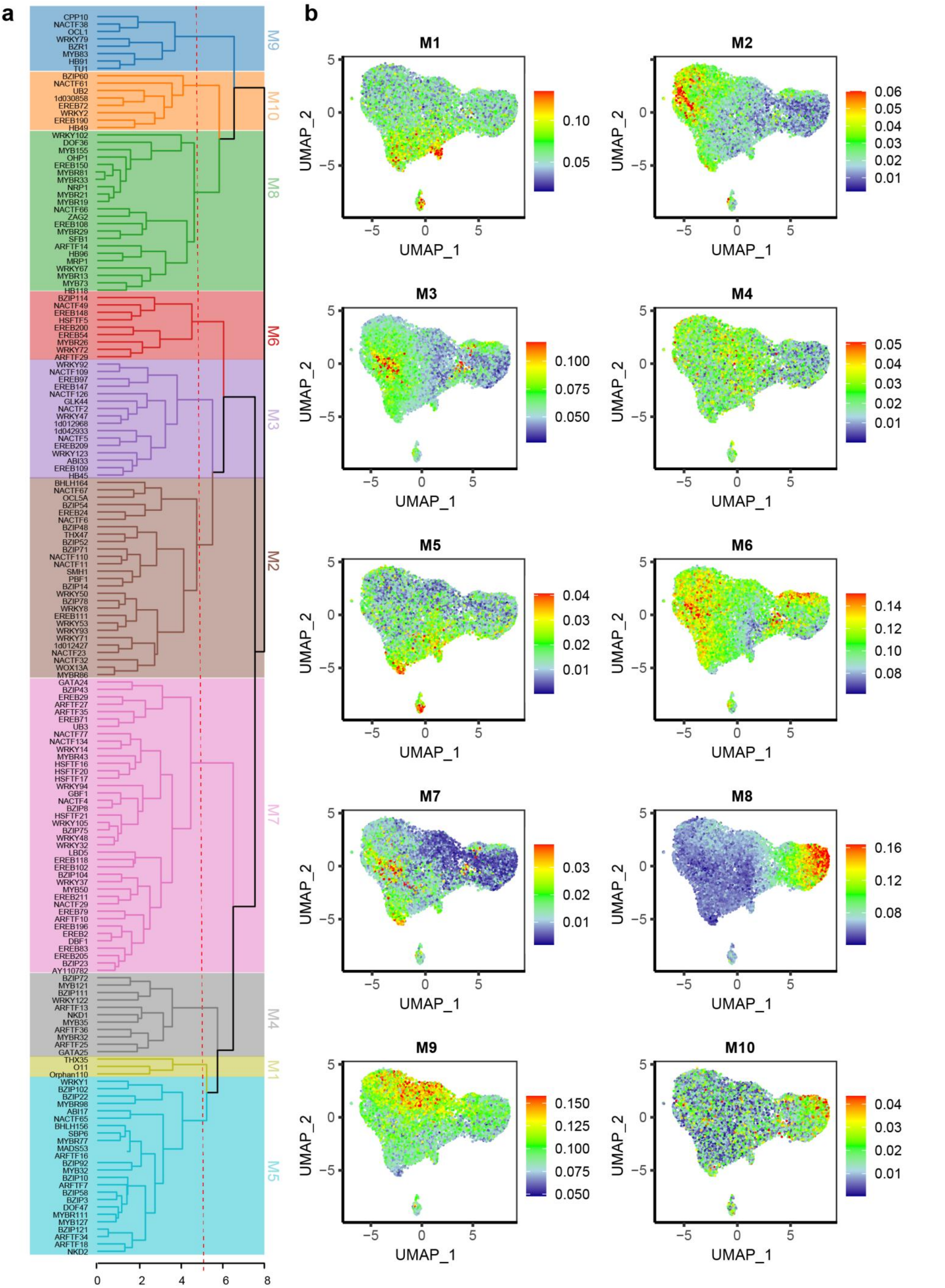

Supplementary Fig. 19. Activities of regulon modules in each cell cluster. a, Hierarchical clustering map of regulons according to the CSI score (set to 5 as a cutoff). **b,** Average activity scores of 10 regulon modules mapped on the UMAP plots.

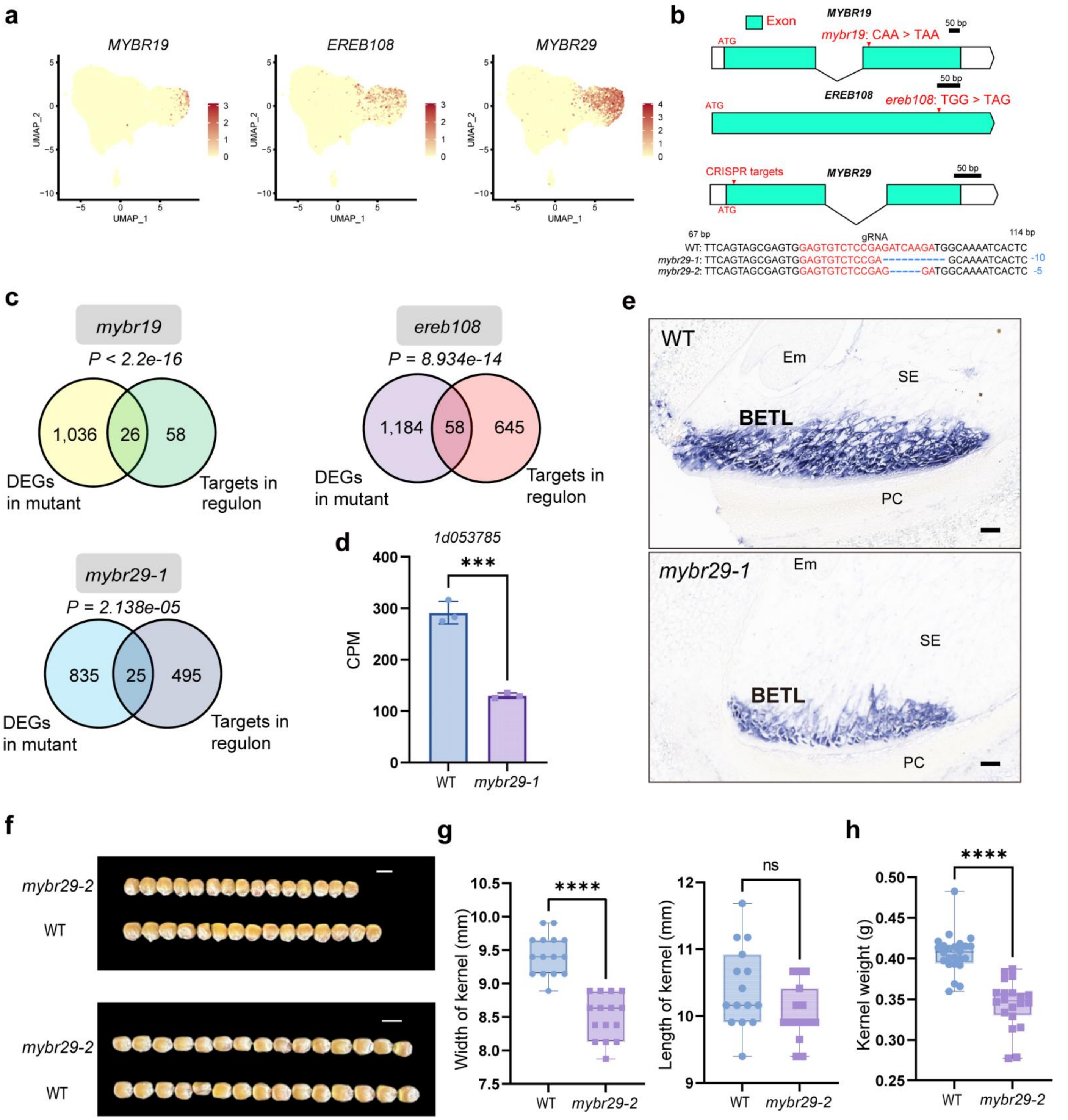

Supplementary Fig. 20. Functional validation of MYBR19, MYBR29 and EREB108 by CRISPR-cas9 mutant and EMS mutant. a, Expression patterns of *MYBR29*, *MYBR19* and *EREB108* in UMAP plots. Color scale represents normalized expression levels. **b,** An early stop codon was introduced in the *MYBR19* and *EREB108* exon region. Schematic diagram of the gene model and mutations identified in two *MYBR29* alleles. **c,** The Venn diagram illustrates the number of overlaps between the differentially expressed genes (DEGs) of three mutant types and the target genes within the regulon. The Fisher's exact test was performed to calculate the *P* value. **d,** Differential expression analysis using bulk RNA-seq suggested that the expression of *1d053785* was significantly decreased in mutant. Error bars indicate ±SEM (n = 3 biologically independent samples). ***, $P < 0.001$. Two-tailed student’s t-test. No adjustments were made for multiple

comparisons test. CPM, Counts per million. **e,** mRNA in situ hybridization results of *1d053785* between WT and *mybr29-1*; scale bars, 100 μm. Embryo (EM), starch endosperm (SE), basal endosperm transfer layer (BETL), placento-chalazal region (PC). Experiments were repeated three times with similar results. **f,** Phenotypic features of the maize *mybr29-2* mutant. Scale bars, 1 cm. **g,** Kernel length and width of WT and *mybr29-2* mature kernels. n = 15 kernels in WT, n = 15 kernels in *mybr29-2*. ****, $P < 0.0001$. Two-tailed student's t-test. No adjustments was made for multiple comparisons test. Box plots indicate median (middle line), 25th, 75th percentile (box) and 5th and 95th percentile (whiskers) as well as all data (single points). **h,** Kernel weights of WT and *mybr29-2* mature kernels. n = 24 kernels in WT, n = 20 kernels in *mybr29-2*. ****, $P < 0.0001$. Two-tailed student's t-test. No adjustments were made for multiple comparisons test. Box plots indicate median (middle line), 25th, 75th percentile (box) and 5th and 95th percentile (whiskers) as well as all data (single points). Source data are provided as a Source Data file.
