# Supplementary material for: Decoding the gene regulatory network of endosperm differentiation in maize
Source: Nat Commun. 2024 Jan 2;15:34. doi: 10.1038/s41467-023-44369-7 (PMC10762121; doi:10.1038/s41467-023-44369-7)
Supplement: Supplementary file 3 — Description of Additional Supplementary Files [file 41467_2023_44369_MOESM3_ESM.pdf]

Description of Additional Supplementary Files:

Supplementary Data 1: Statistic of scRNA-seq.

Supplementary Data 2: A view of 3D UMAP Plot.

Supplementary Data 3: List of cell cycle-related genes.

Supplementary Data 4: List of more strongly expressed genes in each cell cluster.

Supplementary Data 5: Enrichment of gene ontology (GO) terms of differentially expressed genes in each cluster.

Supplementary Data 6: List of phytohormone-related and selected functional genes.

Supplementary Data 7: List of differentially expressed genes across clusters within each cell type.

Supplementary Data 8: List of CCTS genes at 6 and 7 days after pollination (DAP) across all clusters.

Supplementary Data 9: Gene sets utilized for calculating cell differentiation scores.

Supplementary Data 10: Top 50 differentiation-related genes predicted by CytoTRACE analysis.

Supplementary Data 11: Quality statistics table for transcription factors profiled by ampDAP-seq.

Supplementary Data 12: Topology analysis of the top-ranked gene regulatory network.

Supplementary Data 13: Complete target gene list of 181 regulons.

Supplementary Data 14: Enriched gene ontology (GO) terms of target genes in each regulon.

Supplementary Data 15: Target genes of O11, BZIP22, and MRP1 used for network evaluation.

Supplementary Data 16: Regulon specificity score (RSS) for each regulon across 12 cell clusters.

Supplementary Data 17: Average regulon activity score (RAS) for each regulon in 12 cell clusters.

Supplementary Data 18: Connection specificity index (CSI) for 168 retained regulons.

Supplementary Data 19: Regulon activity score (RAS) of 10 regulon modules in 12 cell clusters.

Supplementary Data 20: Module assignment details for each regulon.

Supplementary Data 21: Target number and threshold parameters utilized in AUCell for each regulon.

Supplementary Data 22: Pearson correlation coefficient (PCC) analysis between cell clusters based on regulon activity score (RAS).

Supplementary Data 23: Differentially expressed genes (DEGs) identified in the ereb108 mutant.

Supplementary Data 24: DEGs Identified in the mybr19 mutant.

Supplementary Data 25: DEGs Identified in the mybr29-1 mutant.

Supplementary Data 26: List of primer sequences used in this study.

Supplementary Data 27: Gene symbol and annotation table.
