# Supplementary material for: Decoding the gene regulatory network of endosperm differentiation in maize
Source: Nat Commun. 2024 Jan 2;15:34. doi: 10.1038/s41467-023-44369-7 (PMC10762121; doi:10.1038/s41467-023-44369-7)
Supplement: Supplementary file 31 — Reporting Summary [file 41467_2023_44369_MOESM31_ESM.pdf]

nature portfolio

Corresponding author(s): Rentao Song, Zeyang Ma

Last updated by author(s): Dec 3, 2023

Reporting Summary

Nature Portfolio wishes to improve the reproducibility of the work that we publish. This form provides structure for consistency and transparency in reporting. For further information on Nature Portfolio policies, see our Editorial Policies and the Editorial Policy Checklist.

Statistics

For all statistical analyses, confirm that the following items are present in the figure legend, table legend, main text, or Methods section.

n/a	Confirmed	
[ ]	[x]	The exact sample size (*n*) for each experimental group/condition, given as a discrete number and unit of measurement
[x]	[ ]	A statement on whether measurements were taken from distinct samples or whether the same sample was measured repeatedly
[ ]	[x]	The statistical test(s) used AND whether they are one- or two-sided<br>*Only common tests should be described solely by name; describe more complex techniques in the Methods section.*
[x]	[ ]	A description of all covariates tested
[ ]	[x]	A description of any assumptions or corrections, such as tests of normality and adjustment for multiple comparisons
[ ]	[x]	A full description of the statistical parameters including central tendency (e.g. means) or other basic estimates (e.g. regression coefficient) AND variation (e.g. standard deviation) or associated estimates of uncertainty (e.g. confidence intervals)
[ ]	[x]	For null hypothesis testing, the test statistic (e.g. *F*, *t*, *r*) with confidence intervals, effect sizes, degrees of freedom and *P* value noted<br>*Give P values as exact values whenever suitable.*
[x]	[ ]	For Bayesian analysis, information on the choice of priors and Markov chain Monte Carlo settings
[x]	[ ]	For hierarchical and complex designs, identification of the appropriate level for tests and full reporting of outcomes
[ ]	[x]	Estimates of effect sizes (e.g. Cohen's *d*, Pearson's *r*), indicating how they were calculated

Our web collection on statistics for biologists contains articles on many of the points above.

Software and code

Policy information about availability of computer code

Data collection	No software was used.
Data analysis	All custom code and scripts generated for this project can be accessed: https://github.com/Huo-qiang/maize-endosperm-scRNA-seq.<br>Ref: Huo, Q & Ma Z. Decoding the gene regulatory network of endosperm differentiation in maize, maize-endosperm-scRNA-seq, https://10.5281/zenodo.10208558 (2023).<br>Other software used:<br>Trimmomatic (version 0.39); bowtie2 (version: 2.4.1); Picard (version 2.25.6); GEM (version 3.4); deepTools (version 3.5.1); Integrative Genomics Viewer (IGV) (version 2.10.0); GenomicFeatures (version 1.44.0); ChIPseeker (version 1.28.3); meme-chip (version 5.3.3); bedtools (version 2.30.0); STAR (version: 2.7.5c); featureCounts (version 2.0.1); Cell Ranger 4.0 (10x Genomics); Seurat (version 4.0.3); gprofiler2 (version 0.2.0); Monocle2 (version 0.2.1); CytoTRACE (version 0.3.3); edgeR (version 3.34.0);UCell (version 1.0.0); GRNboot2 (version 0.1.6); Cytoscape (version 3.8.0); AUCell (version 1.14.0); philentropy (version 0.5.0); ggplot2 (version 3.3.5).

For manuscripts utilizing custom algorithms or software that are central to the research but not yet described in published literature, software must be made available to editors and reviewers. We strongly encourage code deposition in a community repository (e.g. GitHub). See the Nature Portfolio guidelines for submitting code & software for further information.

Data

Policy information about availability of data

All manuscripts must include a data availability statement. This statement should provide the following information, where applicable:

- Accession codes, unique identifiers, or web links for publicly available datasets
- A description of any restrictions on data availability
- For clinical datasets or third party data, please ensure that the statement adheres to our policy

The scRNA-seq, bulk RNA-seq and DAP-seq source data are deposited at the Gene Expression Omnibus website (https://www.ncbi.nlm.nih.gov/geo/) under the SuperSeries accession number GSE201701.

Research involving human participants, their data, or biological material

Policy information about studies with human participants or human data. See also policy information about sex, gender (identity/presentation), and sexual orientation and race, ethnicity and racism.

Reporting on sex and gender	N/A
Reporting on race, ethnicity, or other socially relevant groupings	N/A
Population characteristics	N/A
Recruitment	N/A
Ethics oversight	N/A

Note that full information on the approval of the study protocol must also be provided in the manuscript.

Field-specific reporting

Please select the one below that is the best fit for your research. If you are not sure, read the appropriate sections before making your selection.

☒ Life sciences ☐ Behavioural & social sciences ☐ Ecological, evolutionary & environmental sciences

For a reference copy of the document with all sections, see nature.com/documents/nr-reporting-summary-flat.pdf

Life sciences study design

All studies must disclose on these points even when the disclosure is negative.

Sample size	For scRNA-seq, approximately 20,000 cell were loaded for each sample.
Data exclusions	Data from scRNA-seq that did not meet quality thresholds were not included in the analysis. Only cells have over 500 but less than 7,500 features (genes), over 1,000 counts (sequence reads), and log2(feature/count) greater than 0.8 were considered high quality and kept for subsequent analyses.
Replication	For scRNA-seq of sample at 6 DAP, we performed one replicate. For scRNA-seq of samples at 7 DAP, we performed three biological replicates. All experiments of mutant phenotype validations were used at least three biological replicates.
Randomization	For mutant phenotype experiments, all samples were collected by randomly selecting kernels from the same ear of each heterozygous plant.
Blinding	Not applicable - It was not applicable based on the experiment design.

Reporting for specific materials, systems and methods

We require information from authors about some types of materials, experimental systems and methods used in many studies. Here, indicate whether each material, system or method listed is relevant to your study. If you are not sure if a list item applies to your research, read the appropriate section before selecting a response.

Materials & experimental systems

n/a	Involved in the study
☐	☒ Antibodies
☒	☐ Eukaryotic cell lines
☒	☐ Palaeontology and archaeology
☒	☐ Animals and other organisms
☒	☐ Clinical data
☒	☐ Dual use research of concern
☐	☒ Plants

Methods

n/a	Involved in the study
☒	☐ ChIP-seq
☒	☐ Flow cytometry
☒	☐ MRI-based neuroimaging

Antibodies

Antibodies used	Anti-Digoxigenin-AP, Fab fragments,from sheep, Roche Diagnostics(Made in Mannheim, Germany), REF 11093274910, LOT 16646821.
Validation	From manufacture's website:"Analysis Note: Cross reactivity to digitoxin and digitoxigenin: <1 %. No cross reactivity with other human estrogen or androgen steroids, e.g. estradiol or testosterone. Cross reactivity with digoxin: not known. Conjugate does not bind to itself at all. Normally one molecule of the conjugate binds to one molecule digoxigenin, although ther are two possible binding sites for digoxigenin. Nonspecific binding to RNA is not expected."

Dual use research of concern

Policy information about dual use research of concern

Hazards

Could the accidental, deliberate or reckless misuse of agents or technologies generated in the work, or the application of information presented in the manuscript, pose a threat to:

No	Yes
☒	☐ Public health
☒	☐ National security
☒	☐ Crops and/or livestock
☒	☐ Ecosystems
☒	☐ Any other significant area

Experiments of concern

Does the work involve any of these experiments of concern:

No	Yes
☒	☐ Demonstrate how to render a vaccine ineffective
☒	☐ Confer resistance to therapeutically useful antibiotics or antiviral agents
☒	☐ Enhance the virulence of a pathogen or render a nonpathogen virulent
☒	☐ Increase transmissibility of a pathogen
☒	☐ Alter the host range of a pathogen
☒	☐ Enable evasion of diagnostic/detection modalities
☒	☐ Enable the weaponization of a biological agent or toxin
☒	☐ Any other potentially harmful combination of experiments and agents

Plants

Seed stocks	Maize (Zea mays L.) B73 inbred line seeds were come from Maize Genetics Cooperation-Stock Center and grown at Shang Zhuang Beijing (40° N, 116° E) during summer in 2020. The mybr19 and ereb108 mutants seeds were purchased from a EMS mutant library (http://elabcaas.cn/memd)
Novel plant genotypes	We generated mybr29 CRISPR-Cas9 transgenic lines using the simplex strategy. Specifically, the 20-bp target editing sequence is located within the first exon of MYBR29 (GAGTGTCTCCGAGATCAAGA). Agrobacterium-mediated maize transformation was employed. To ensure genetic stability, both mybr29-1 and mybr29-2 were backcrossed to the B104 genetic background for at least two generations.
Authentication	*Describe any authentication procedures for each seed stock used or novel genotype generated. Describe any experiments used to assess the effect of a mutation and, where applicable, how potential secondary effects (e.g. second site T-DNA insertions, mosiacism, off-target gene editing) were examined.*
