# Supplementary material for: Decoding the gene regulatory network of endosperm differentiation in maize
Source: Nat Commun. 2024 Jan 2;15:34. doi: 10.1038/s41467-023-44369-7 (PMC10762121; doi:10.1038/s41467-023-44369-7)
Supplement: Supplementary file 2 — Peer Review File [file 41467_2023_44369_MOESM2_ESM.pdf]

nature portfolio

Peer Review File

Decoding the gene regulatory network of endosperm differentiation in maize

Open Access This file is licensed under a Creative Commons Attribution 4.0 International License, which permits use, sharing, adaptation, distribution and reproduction in any medium or format, as long as you give appropriate credit to the original author(s) and the source, provide a link to the Creative Commons license, and indicate if changes were made. In the cases where the authors are anonymous, such as is the case for the reports of anonymous peer reviewers, author attribution should be to 'Anonymous Referee' followed by a clear attribution to the source work. The images or other third party material in this file are included in the article's Creative Commons license, unless indicated otherwise in a credit line to the material. If material is not included in the article's Creative Commons license and your intended use is not permitted by statutory regulation or exceeds the permitted use, you will need to obtain permission directly from the copyright holder. To view a copy of this license, visit http://creativecommons.org/licenses/by/4.0/.

Editorial Note: This manuscript has been previously reviewed at another journal that is not operating a transparent peer review scheme. This document only contains reviewer comments and rebuttal letters for versions considered at *Nature Communications*.

Reviewer #1 (Remarks to the Author):

The authors have addressed all my concerns with this revised manuscript

Reviewer #2 (Remarks to the Author):

In this manuscript, Yuan et al. leverage single-cell RNA-seq and DAP-seq to explore the regulatory mechanism of maize endosperm cell fate differentiation by profiling 6DAP, 7DAP, and 8DAP maize endosperm. The author presented a rebuttal letter to the previous reviewer's comments. I have a few comments.

First, the 8DAP dataset quality is concerning. The fraction of reads in cells was only around 30%, which is usually low. The low fraction reads in cells usually indicated that the samples very likely have high ambient RNA, which might be caused by burst protoplast or other cell preparation problems. High ambient RNA will blur the clustering. Even if the authors applied harsh filtration as explained in the rebuttal letter, the ambient RNA will remain in the cells that are kept after filtration. This could be the reason why cell types AL and SE are not fully differentiated by 8 DAP. In the rebuttal letter, the author used two examples both published in Plant Biotechnol. J. (Wang et al., 2022 and Qin et al., 2022) to justify that such quality datasets could be acceptable. However, only Qin et al. 2022 published four out of six samples that have similar (around 30%) low fraction reads in cells. Another reason for the low fraction of reads in cells could be cell-calling heuristic did not apply well for 8DAP when CellRanger is calling cells, which can be interpreted from the ranked barcode plot in the Cell Ranger web_summary.html file. A side-by-side comparison of the ranked barcode plot of all 6DAP, 7DAP, and 8DAP may tell the difference. This reason is unlikely unless the authors have biological evidence to justify that 8DAP cells indeed have higher variation in RNA content (in other words, more cells with lower RNA content).

Second, given the concern of 8DAP dataset quality, the discussion of cellular differentiation (figure 3) might need to be re-evaluated after improving it. The author also showed in supplementary figure 13.b that the 6DAP and 7DAP stages have similar differentiation scores. While 8DAP is distinct from both 6DAP and 7DAP. Only 6DAP and 8DAP datasets were used to calculate the cellular differentiation score based on the original code and methods. It's unclear if the author used bulk RNA-seq for these stages, or using the pseudobulk of single-cell RNA-seq datasets. The differential expression analysis mentioned in lines 259 – 261 for these three stages is for each cell type. However, the "Gene expression pattern analysis" in the method section (line 704) didn't clearly describe how each cell type was calculated separately. Did the author use pseudobulk analysis of each cell type (if so, then how did the author process 6DAP because it only has one replicate), or consider each cell as one replicate (if so, how did the author address the extreme p-value, and how p_val_adj_cluster was calculated based on Bonferroni correction)?

Minor comments:

The authors did several rounds of cell removal and re-clustering. In Supplementary Figure 3.a, there are a total of 18 clusters, including cells that are enriched in mitochondria (cluster 16), embryo cells (cluster 17), cells that respond to stimulus stress (cluster 13), and nucellus cells (cluster 14). These cells were removed when presenting the cells in Figure 1b. Then in Figure 2a, cycling cells were further removed. Did the author re-normalize and re-scale the data using cells after removal was done each round? Based on the original codes on GitHub, it seems the authors did not do it. The authors need to clearly describe how these cells were processed sequentially in the method section. Please also add English translations for some Chinese languages written in the code.

In supplementary data 2, and method section lines 663-666, I don't think it's accurate to simply use 1-1 ortholog approach to identify maize cycling orthologous genes from the rice study. This will largely miss many maize cycling genes, and it's indeed a known hurdle in the plant single-cell analysis field for getting a complete list of cell cycle genes. The author needs to discuss the limitations of this part of the analysis in the manuscript.

Reviewer #3 (Remarks to the Author):

The authors have adequately addressed my concerns and comments raised in my last review.

We thank the reviewers for their suggestions. We have revised the manuscript carefully. The detailed point-by-point responses to the reviewers' comments are highlighted below, with quoted text from the manuscript provided in *blue and italic*.

Reviewer #1 (Remarks to the Author):

The authors have addressed all my concerns with this revised manuscript

Response: Thank you for your appreciation!

Reviewer #2 (Remarks to the Author):

In this manuscript, Yuan et al. leverage single-cell RNA-seq and DAP-seq to explore the regulatory mechanism of maize endosperm cell fate differentiation by profiling 6DAP, 7DAP, and 8DAP maize endosperm. The author presented a rebuttal letter to the previous reviewer's comments. I have a few comments.

First, the 8DAP dataset quality is concerning. The fraction of reads in cells was only around 30%, which is usually low. The low fraction reads in cells usually indicated that the samples very likely have high ambient RNA, which might be caused by burst protoplast or other cell preparation problems. High ambient RNA will blur the clustering. Even if the authors applied harsh filtration as explained in the rebuttal letter, the ambient RNA will remain in the cells that are kept after filtration. This could be the reason why cell types AL and SE are not fully differentiated by 8 DAP. In the rebuttal letter, the author used two examples both published in Plant Biotechnol. J. (Wang et al., 2022 and Qin et al., 2022) to justify that such quality datasets could be acceptable. However, only Qin et al. 2022 published four out of six samples that have similar (around 30%) low fraction reads in cells. Another reason for the low fraction of reads in cells could be cell-calling heuristic did not apply well for 8DAP when CellRanger is calling cells, which can be interpreted from the ranked barcode plot in the Cell Ranger web_summary.html file. A side-by-side comparison of the ranked barcode plot of all 6DAP, 7DAP, and 8DAP may tell the difference. This reason is unlikely unless the authors have biological evidence to justify that 8DAP cells indeed have higher variation

in RNA content (in other words, more cells with lower RNA content).

Response: Thank you for your comments.

Firstly, it is important to note that our previous 6-8 DAP samples were collected under the same environmental condition. Repeating the profiling of 8 DAP cells alone might compromise the overall consistency and integrity of the entire original dataset. Secondly, from a technical perspective, we indeed attempt to reproduce the profiling of endosperm samples during the late differentiation stage. However, we observed that the "fraction of reads in cells" QC metric remained lower as compared to the 6 and 7 DAP samples. This might be due to the possibility that some endosperm cells had increased in size after 8 DAP, making them more prone to burst when captured by the oil droplets.

Nevertheless, considering these factors, in order to completely eliminate the possibility of any potentially adverse impact on our conclusions, we chose to exclude the 8 DAP samples and re-performed all the analyses. We were pleased to find that the results closely mirror our previous findings, except for a few changes:

a). The number of average expressed genes per cell is now increased from 1,484 to 2,005.

b). The cell clustering results remain the same, but the marker gene lists for each cell cluster have slightly differences. New plots of marker genes (*1d053108* for BETL, *TAR1* for SE, and *PEBP11* for EAS) have been added to annotate their cell type identities.

c). The conclusion that AL and SE cells are not fully differentiated at 8 DAP is no longer valid. And we have changed text related to the differentiation status of AL and SE cells in Lines 169-170 of our revised manuscript:

"This suggests a close relationship between the AL and the SE, and implies that they are not fully differentiated at 7 DAP."

d). The panels with 8 DAP samples in previous Fig 3 were changed along with their related descriptions. Thus, "the significantly decreased cell number of BETL and increased cell number of EAS at 8 DAP" was removed. (See more detailed information in the response below)

We believe that our new results provide further support for the validity of our other prior conclusions.

Second, given the concern of 8DAP dataset quality, the discussion of cellular differentiation (figure 3) might need to be re-evaluated after improving it. The author also showed in supplementary figure 13.b that the 6DAP and 7DAP stages have similar differentiation scores. While 8DAP is distinct from both 6DAP and 7DAP. Only 6DAP and 8DAP datasets were used to calculate the cellular differentiation score based on the original code and methods. It's unclear if the author used bulk RNA-seq for these stages, or using the pseudobulk of single-cell RNA-seq datasets. The differential expression analysis mentioned in lines 259 – 261 for these three stages is for each cell type. However, the "Gene expression pattern analysis" in the method section (line 704) didn't clearly describe how each cell type was calculated separately. Did the author use pseudobulk analysis of each cell type (if so, then how did the author process 6DAP because it only has one replicate), or consider each cell as one replicate (if so, how did the author address the extreme p-value, and how p_val_adj_cluster was calculated based on Bonferroni correction)?

Response: Thank you for these comments.

-- For the results of cellular differentiation, we have generated a new figure after removing the 8 DAP samples (Fig. 1; also as shown in Fig. 3a-f of the revised manuscript) and changed the text related to it in Lines 249-262 of the revised manuscript:

"UMAP plots showed that all clusters were continuously detected from 6 and 7 DAP (Fig. 3a). However, the sizes of some cell clusters changed significantly across this time period (Fig. 3b). For example, the proportional cell number of ESR cluster 15 dramatically increased (from 0.1% at 6 DAP to over 1.2% at 7 DAP) (Fig. 3b). In addition, differential expression analysis revealed a cohort of genes with temporally-specific enrichment in each cell cluster, indicating that these clusters were undergoing a vigorous differentiation process (Fig. 3c, Supplementary Data 7 and see Methods). We defined these as cell-cluster-time-specific (CCTS) genes. For

example, expression of the defense related genes Defensin-like protein (DEF)1 and DEF2 of AL clusters 0, 3, 4, and 7 increased from 6 to 7 DAP (Fig. 3c, Supplementary Data 7). Interestingly, the temporal gene expression patterns of each cell cluster corresponded to the cell cluster identity, a large percentage of CCTS genes being also cluster-representative markers (Fig. 3c and Supplementary Data 7). For example, Maternally expressed gene (MEG)1 and MEG3 were CCTS genes and also identified BETL cluster 2 markers (Fig. 3c).”

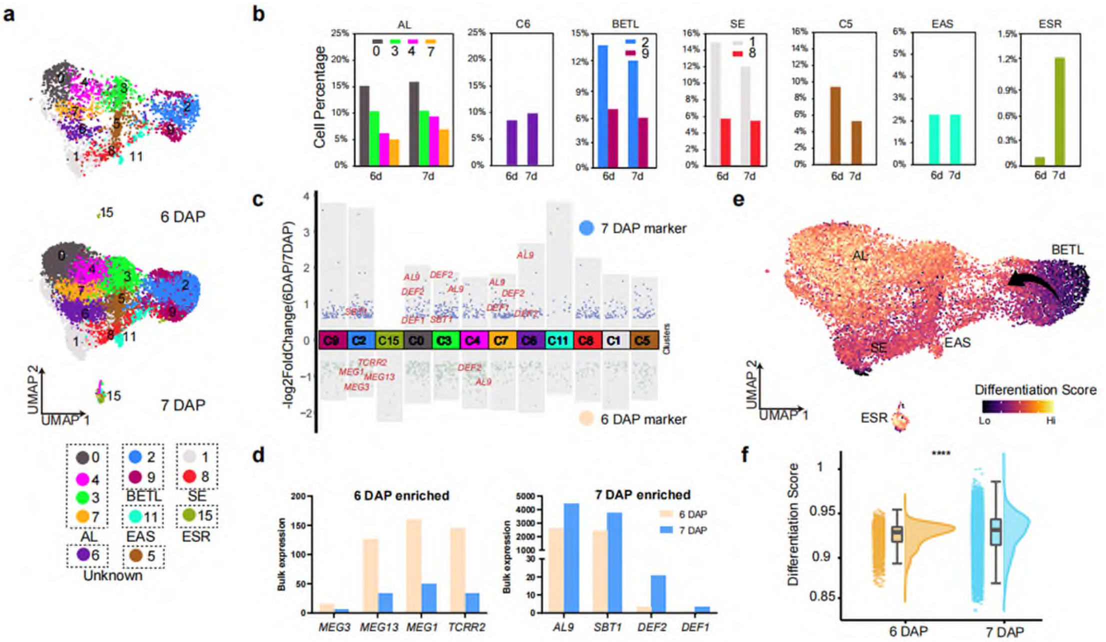

Fig. 1 Transcriptome dynamics at the transition stage during endosperm differentiation. a) Visualization of five endosperm cell types by UMAP plots. b) Bar charts showing the ratio of cell number of different cell clusters at 6 and 7 DAP, colored as in (a). c) Differential gene expression analysis showing 6 DAP and 7 DAP marker genes across all 12 clusters. d) Expression levels of selected marker genes at 6 and 7 DAP from bulk RNA-seq. e) Trajectories of developmental “pseudotime” in each cell cluster mapped onto the same UMAP depicted in (a); the differentiation score is calculated based on the stage-specific genes identified from bulk RNA-seq. f) Raincloud plot showing the cell differentiation score of 6 and 7 DAP. ****, $P < 0.0001$; T-test.

-- Regarding the calculation of the cellular differentiation score, we previously utilized STEM (version 1.3.13) with the bulk RNA-seq data at different time points, each with three biological replicates. In our revised manuscript, we have employed edgeR (version 3.34.0) to calculate marker genes for the 6 DAP and 7 DAP samples (Fig. 2; also as shown in Supplemental Fig. 12 of the revised manuscript). The

generation of the "Cellular differentiation score" has been described in the methods section (In Line 704-712 of the revised manuscript). Furthermore, we observed that the new cellular differentiation score for 7 DAP is significantly higher than that at 6 DAP. (Fig. 3; also as shown in Fig. 3f of the revised manuscript)

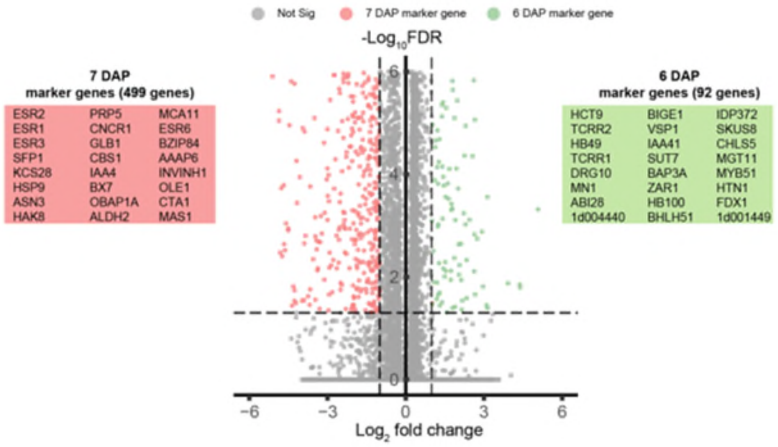

Fig. 2 Identification of marker genes at 6 and 7 DAP. Differentially expressed genes between 6 DAP and 7 DAP from bulk RNA-seq.

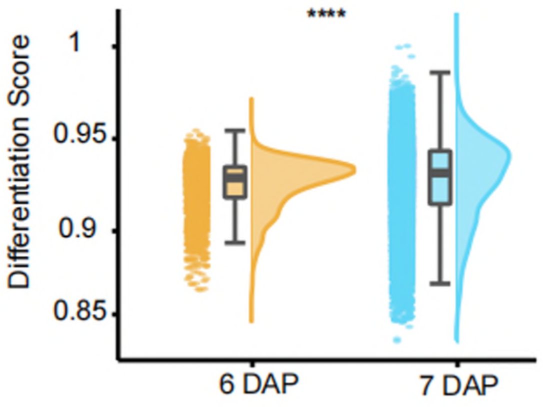

Fig. 3 Raincloud plot showing the cell differentiation score of 6 and 7 DAP. ****, $P < 0.0001$; T-test.

-- The temporal differential expression analysis for each cell type were calculated by FindAllMarkers() function of Seurat package. Each cell was treated as one replicate. Regarding the handling of extreme p-values and the calculation of p_val_adj, the FindAllMarkers() function employs the Bonferroni correction method to address extreme p-values, see:

(https://github.com/satijalab/seurat/blob/master/R/differential_expression.R).

When performing differential gene expression analysis, raw p-values are generated to assess the significance of differences between genes in different cell groups. Then the Bonferroni correction is applied. This correction adjusts the raw p-values by multiplying them by the total number of comparisons, effectively controlling the error rate associated with multiple testing. If genes are to be compared between different cell groups, the total number of comparisons will be the number of genes multiplied by the number of cell groups. The result is "p_val_adj", representing the Bonferroni-corrected p-values, which is used to gauge the significance of each gene across cell groups (cells in a cell type at different time points in our case).

Minor comments:

The authors did several rounds of cell removal and re-clustering. In Supplementary Figure 3.a, there are a total of 18 clusters, including cells that are enriched in mitochondria (cluster 16), embryo cells (cluster 17), cells that respond to stimulus stress (cluster 13), and nucellus cells (cluster 14). These cells were removed when presenting the cells in Figure 1b. Then in Figure 2a, cycling cells were further removed. Did the author re-normalize and re-scale the data using cells after removal was done each round? Based on the original codes on GitHub, it seems the authors did not do it. The authors need to clearly describe how these cells were processed sequentially in the method section. Please also add English translations for some Chinese languages written in the code.

Response: Thank you for raising this point. We apologies for the lack of clarity in our method description. Indeed, after removing the specific cell populations to create new datasets, we took the necessary steps for re-clustering our data. Additionally, we have also updated the relevant code on GitHub with English annotation and added this description in the Methods section (Lines 666-668 of the revised manuscript).

In supplementary data 2, and method section lines 663-666, I don't think it's accurate to simply use 1-1 ortholog approach to identify maize cycling orthologous genes from the rice study. This will largely miss many maize cycling genes, and it's indeed a known hurdle in the plant single-cell analysis field for getting a complete list of cell cycle genes. The author needs to discuss the limitations of this part of the analysis in the manuscript. [adding discussion about cell cycling genes]

Response: Thank you for the suggestion. We'd like to emphasize that the gene set obtained from rice homologous is just an additional supplement to the reported maize gene set we have used. We have added a discussion for the cell cycle analysis in the discussion section (Lines 481-486 of the revised manuscript).

"In scRNA-seq data, the captured cells can often originate from different cell cycle stages. It is therefore necessary to remove cell cycle effects on clustering from the analysis. To do so, we have collected a number of reported maize cell cycle marker genes and homologs based on a study in rice. However, a small group of cells are still annotated as being in the G2/M phase (Fig 1b), which could be due to the incomplete annotation of cell cycle marker genes."

Reviewer #3 (Remarks to the Author):

The authors have adequately addressed my concerns and comments raised in my last review.

Response: Thank you for your appreciation!

Reviewer #2 (Remarks to the Author):

The authors have addressed all my major concerns with this revised manuscript. Removing problematic datasets is indeed a wise option.

Two very minor comments:

>> The author wrote “It is therefore necessary to remove cell cycle effects on clustering from the analysis” in line 482. I think “necessary” here might be a bit misleading. Large portions of plant single-cell publications keep cells undergoing cell cycles as separate clusters. I would suggest the author simply describe what they have done in the manuscript, “we attempted to remove cell cycle effects on clustering from the analysis”.

>> I would suggest the author add the method about the temporal differential expression analysis for each cell type. Including: Calculated by FindAllMarkers() function of Seurat package; Each cell was treated as one replicate; Employing the Bonferroni correction of p-value

We thank the reviewers for their suggestions. The detailed point-by-point responses to the reviewers' comments are highlighted below, with quoted text from the manuscript provided in *blue and italic*.

Reviewer #2 (Remarks to the Author):

The authors have addressed all my major concerns with this revised manuscript. Removing problematic datasets is indeed a wise option.

Response: Thank you for your appreciation!

Two very minor comments:

>> The author wrote "It is therefore necessary to remove cell cycle effects on clustering from the analysis" in line 482. I think "necessary" here might be a bit misleading. Large portions of plant single-cell publications keep cells undergoing cell cycles as separate clusters. I would suggest the author simply describe what they have done in the manuscript, "we attempted to remove cell cycle effects on clustering from the analysis".

Response: Thank you for the suggestion. We have revised this sentence as suggested in "Discussion" section in Lines 496-497 of the revised manuscript.

>> I would suggest the author add the method about the temporal differential expression analysis for each cell type. Including: Calculated by FindAllMarkers() function of Seurat package; Each cell was treated as one replicate; Employing the Bonferroni correction of p-value

Response: Thank you for the suggestion. We have added this in "Methods" section in Lines 682-685 of the revised manuscript.

"Temporal differential expression for each cell type was analyzed using the Seurat package's FindAllMarkers(Wilcoxon rank-sum test, min.pct = 0.3, logfc.threshold = 0.25) function, treating each cell as a replicate and applying Bonferroni correction for p-value adjustment."
